# AlF_3_ Mediated In‐Situ Cathode Interface Stabilization Enables High‐Rate and Long‐Life Na‐Ion Batteries at Elevated Temperature

**DOI:** 10.1002/advs.202522907

**Published:** 2026-01-14

**Authors:** Ya‐Meng Yin, Qinxia Liu, Zhiyuan Zhang, Weixiao Wang, Cunyuan Pei, Fangyu Xiong, Jinghui Chen, Qinyou An, Cai‐Hong Yang, Dong‐Sheng Li

**Affiliations:** ^1^ Key Laboratory of Inorganic Nonmetallic Crystalline and Energy Conversion Materials College of Materials and Chemical Engineering China Three Gorges University Yichang Hubei China; ^2^ Hubei Three Gorges Laboratory Yichang Hubei China; ^3^ State Key Laboratory of Advanced Technology For Materials Synthesis and Processing Wuhan University of Technology Wuhan China; ^4^ National Engineering Research Center For Magnesium Alloys, College of Materials Science and Engineering Chongqing University Chongqing China

**Keywords:** AlF_3_ coating, high temperature, inorganic‐dominated CEI, Na_3_V_2_(PO_4_)_2_O_2_F

## Abstract

The instability of the cathode electrolyte interface (CEI) under high voltage and elevated temperature poses a major challenge to the practical application of Na_3_V_2_(PO_4_)_2_O_2_F (NVOPF) cathodes in sodium‐ion batteries (SIBs). To overcome this issue, we introduce a dual‐functional AlF_3_ coating that effectively stabilizes the interface while improving bulk electronic conductivity. The AlF_3_‐modified NVOPF@C exhibits exceptional cycling stability at 55°C, maintaining 84.9% capacity after 1000 cycles at 10 C in half cells and 96.8% at 5 C in full cells using hard carbon anodes. Detailed characterization reveals that the AlF_3_ coating promotes the formation of a robust, inorganic‐rich CEI, primarily composed of a NaF/AlF_3_/NaAlF_4_ ternary fluoride composite. This newly constructed CEI layer not only acts as a protective barrier to suppress detrimental interfacial side reactions but also serves as an efficient ionic conductor to facilitate Na^+^ diffusion. In addition, the AlF_3_ coating induces the creation of F─Al─O bridging bonds with surface oxygen groups, which collaborate with carbon nanotubes to establish a highly continuous conductive network that enables efficient electron transfer. These findings underscore the crucial significance of constructing a stable inorganic‐dominated CEI and continuous conductive pathways for developing high‐rate and thermally stable SIBs.

## Graphical abstract

Ultrathin AlF_3_ coating facilitates the formation of an inorganic‐dominated cathode electrolyte interphase (CEI), predominantly composed of NaF/AlF_3_/NaAlF_4_. This CEI layer effectively suppresses detrimental interfacial side reactions while simultaneously enhancing the diffusion kinetics of Na^+^. As a result, the modified NVOPF exhibits outstanding long‐term cycling stability under high rates in both half and full cells, particularly at 55°C.

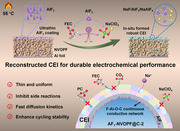

## 1 Introduction

Sodium‐ion batteries (SIBs) are gradually being recognized as a competitive battery technology in the field of large‐scale electrical energy storage systems because of the abundant resources, low cost, high safety, and environmental friendliness [1]. Crucial to SIBs, cathode materials determine the overall battery performance, particularly governing the energy density and high rate cycling stability [2]. Therefore, it is urgent to develop cathode materials with high voltage and fast charging/discharging properties to accelerate the market penetration of SIBs. Polyanionic compounds are considered as strong contenders owing to their high operating potentials induced by polyanion groups and rapid Na ion de/intercalation kinetics due to the robust 3D framework [3]. Typically, the fluorine‐substituted NASICON‐type phosphate material Na_3_V_2_(PO_4_)O_2_F (NVOPF) delivers a theoretical energy density of 500 Wh kg^−1^, attributed to its impressive specific capacity (130 mAh g^−1^) and high working voltage (∼3.9 V vs. Na^+^/Na) [4]. It is comparable to the energy density of LiFePO_4_ (528 Wh kg^−1^) [5], a commercial cathode material widely utilized in commercial lithium‐ion batteries. Despite the above advantages, surface and interface issues caused by in situ formed cathode‐electrolyte interfaces (CEI) on the NVOPF surface fundamentally determine the electrochemical behavior. The instability of derived CEI destroys the cathode bulk structure and causes severe electrolyte depletion, especially at high voltage and elevated temperatures [6]. Particularly, when the battery is operated at high rates, the increased interfacial current density exacerbates internal reactions and directly leads to the degradation of CEI and undesirable side reactions, resulting in continuous performance degradation [7, 8, 9]. Thus, rationally constructing a thermally and mechanically stable CEI layer for NVOPF is urgently required to meet the practical application in harsh environments.

Usually, high temperature conditions generate more organic reduction products on the surface of the cathode and increase its electronic energy level, resulting in a thick and unstable CEI layer [10]. This compromised CEI not only increases the internal resistance of the battery but also exacerbates side reactions, thereby accelerating cathode failure. On this basis, the design of the CEI layer needs to be carefully considered to ensure the fast charge transfer between interfaces and maintain the protection of cathode materials. Studies have demonstrated that CEI enriched with inorganic components (especially NaF) is essential in boosting Na ion transportation and tends to generate robust and dense cover on the cathode surface [11]. Focus is placed on generating fluorine‐containing compounds through rational fluorinated interface engineering to enable excellent electrochemical performance [12, 13]. However, surface fluorinated treatment on CEI upon cycling for cathode materials is extremely difficult. In the commonly used carbonate‐based electrolytes, CEI formation is primarily derived from solvent oxidation or catalyzed decompositions, yielding an organic dominated interphase [14]. By fluorine substitution in carbonate solvents, limited amounts of stable polymer species and NaF can be obtained in CEI during oxidation. Recently, it was revealed that the content of fluorides can be increased via a synergy effect between electrolytes and fluoride coatings, achieved through the deposition of a metal fluoride layer. This newly formed fluorinated interphase modifies charge transfer and improves surface stabilization. Despite these advances, key characteristics such as the thickness, morphology, elemental composition, processing procedure, and their mechanistic influence under high voltage and elevated temperature conditions remain to be elucidated.

**FIGURE 1:**
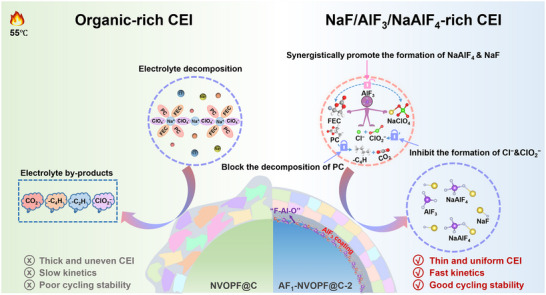
Schematic diagram of CEI formation of NVOPF@C and AF_1_‐NVOPF@C‐2 cathode.

In this study, an ultrathin AlF_3_ interfacial layer measuring about 3 nm was constructed on the surface of carbon nanotubes (CNTs) enhanced NVOPF (NVOPF@C) to improve the high temperature interphase stability. Systematic investigations indicate that the AlF_3_ coating layer undergoes favorable interactions with electrolytes during charge/discharge processes, leading to inorganic‐dominated uniform and dense CEI. As depicted in Figure 1, pristine NVOPF@C experiences side reactions triggered by electrolyte erosion after cycling at elevated temperatures. Fragments such as ─C_4_H, ─C_2_H, and ─ClO_2_ generated during this process deposit on its surface, resulting in thick CEI layers with poor uniformity. In addition, the accompanied CO_2_ release further compromises the integrity of CEI, resulting in sluggish Na ion diffusion kinetics and poor cycling performance. After being decorated with a uniform nanoscale AlF_3_ coating, a portion of the AlF_3_ coating reacts with O^2−^ (from PO_4_^3−^ groups or adsorbed oxygen species) to form F─Al─O bridging intermediates, which act as a chemical transition layer between AlF_3_ and NVOPF@C. This layer not only strengthens the interfacial bonding between the AlF_3_ coating and NVOPF substrate, but also combines with CNTs by the formation of F─Al─O─C to establish a continuous conductive pathway, thereby significantly boosting the surface electrical conductivity. Furthermore, AlF_3_ plays a crucial role in regulating CEI reconstruction. On one hand, partial dissolution of the AlF_3_ coating on the electrolyte side promotes the synergistic reaction between FEC and NaClO_4_, contributing to the in‐situ formation of NaAlF_4_ and NaF. These compounds embedded within the CEI layer as ionic conductors, resulting in a new CEI enriched with ternary fluorides of NaF/AlF_3_/NaAlF_4_. On the other hand, the AlF_3_ coating also suppresses the side reaction of ClO_4_^−^ to inorganic Cl^−^ and ClO_2_^−^, thereby stabilizing the chemical state of Cl and reducing its irreversible loss. Additionally, the AlF_3_ coating acts as a barrier that prevents the electrolyte from interacting with the active material, thus suppressing oxidative decomposition of propylene carbonate (PC) and minimizing the generation of organic fragments which impede Na ion transport. Consequently, the evolved CEI exhibits a uniform, dense, and robust property, which significantly enhances Na ion diffusion and substantially improves the high rate cycling stability.

## 2 Results and Discussion

**FIGURE 2:**
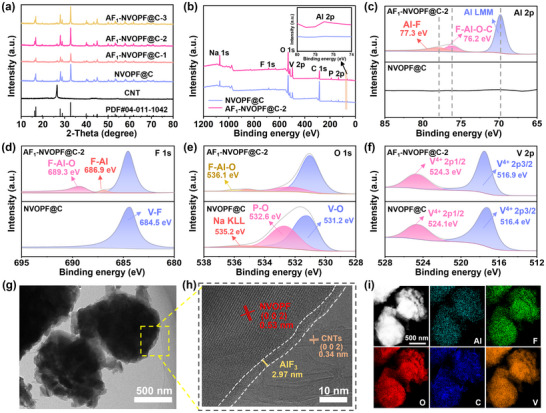
a) XRD patterns of NVOPF@C, AF_1_‐NVOPF@C‐1, AF_1_‐NVOPF@C‐2, and AF_1_‐NVOPF@C‐3. b) XPS full spectrum, c‐f) Al 2p, F 1s, O 1s and V 2p XPS spectrum of NVOPF@C and AF_1_‐NVOPF@C‐2. g) TEM, h) HRTEM images, i) HAADF image and the corresponding elemental mappings of AF_1_‐NVOPF@C‐2.

AlF_3_‐modified Na_3_V_2_(PO_4_)_2_O_2_F was synthesized via a typical hydrothermal synthesis followed by a simple co‐precipitation process. CNTs were introduced to solve the problem of poor electronic conductivity of NVOPF. According to the thermogravimetric analysis results (Figure S1), the actual incorporation of CNTs is 7 wt% of the NVOPF samples. AlF_3_ coating was performed on this basis to improve the interfacial stability during cycling. In the post‐coating process, it was found that the direct wet coating of AlF_3_ (pH = 4 of the coating solution) leads to the cleavage of NVOPF, resulting in damage to its original morphology (Figure S2a–c). Therefore, hydrofluoric acid (HF) or ammonium hydroxide (NH_3_·H_2_O) was used to adjust the pH value to 1, 7, and 11 (Figure S2d–f). However, the original morphology of NVOPF and a uniform AlF_3_ coating (Figure S3) can be maintained only at pH = 7. Figure 2a shows that all the X‐ray diffraction (XRD) peaks of the samples are consistent to the standard tetragonal Na_3_V_2_(PO_4_)_2_O_2_F (JCPDS card No. 04‐011‐1042) and CNTs, with high crystallinity and no other impurity peaks. The AlF_3_ coating has no significant impact on the purity of NVOPF, however, it was not detected [15]. To further substantiate that AlF_3_ was successfully coated on the surface of AF_1_‐NVOPF@C‐2, samples with higher AlF_3_ coating (5% and 10%) and as well as pure AlF_3_ (synthesized without NVOPF and CNTs) were prepared and analyzed by XRD. As shown in (Figure S4), distinct diffraction peaks corresponding to the β‐AlF_3_ phase (JCPDS card No.00‐31‐0011) are observed for both the high‐content AlF_3_ coated samples and the pure AlF_3_. Meanwhile, the thickness of the coating layer could strike a balance between protective properties and functionality [16]. Excessive coating thickness hinders Na ion diffusion and increases internal resistance, whereas insufficient thickness fails to inhibit side reactions and ultimately compromising electrochemical performance. Therefore, the content of the AlF_3_ coating was controlled at 0.5%, 1% and 2% to obtain the optimal amount of coating. Figure S5 shows that the sample with 1% AlF_3_ coating displays the best rate performance. Therefore, all the subsequent comparisons were performed between AF_1_‐NVOPF@C‐2 (AlF_3_ coating solution with pH = 7 and 1% coating quality) and the original NVOPF@C.

The composition of the surface coating was further elucidated by X‐ray photoelectron spectroscopy (XPS) analysis. The wide XPS spectra of AF_1_‐NVOPF@C‐2 and NVOPF@C confirm the presence of Na, V, O, C, P, and F (Figure 2b). Notably, a clear detection of an additional Al peak in AF_1_‐NVOPF@C‐2 confirms the successful coating of AlF_3_ on NVOPF@C. In the Raman spectrum of AF_1_‐NVOPF@C‐2 (Figure S6), the characteristic peak associated with the AlF_3_ R3C space group is observed at 160 cm^−1^, demonstrating the decoration of the AlF_3_ layer [17]. Quantitative analysis of AlF_3_ in combination with ICP‐OES measurements shows that the actual content is 0.7% (Table S1). The typical existence of Al−F peak is confirmed in the Al 2p peak of AF_1_‐NVOPF@C‐2 [18]. Besides, the F─Al─O─C bond is surprisingly detected at 76.2 eV (Figure 2c) [19]. The formation mechanism includes two steps. Initially, the surface of NVOPF@C may contain residual oxygen groups (e.g., O^2–^ in PO_4_^3–^ or adsorbed oxygen species), which react with part of the AlF_3_ coating to form F─Al─O bridging intermediates [20]. Subsequently, these intermediates interact with the CNTs and ultimately generate F─Al─O─C hybrid bonds. The existence of these bonds is also verified through the deconvolution of C 1s XPS spectra (Figure S7). Compared to NVOPF@C (286.1 eV), the C─O peak in AF_1_‐NVOPF@C‐2 shifts to a lower binding energy (285.6 eV). This shift signifies an electron‐rich environment around the carbon atoms, resulting from an electronic interaction within the newly formed Al─O─C structure [21]. The characteristic peak at 689.3 eV in the F 1s peak (Figure 2d) and 536.1 eV in the O 1s peak (Figure 2e) together further demonstrate the formation of F─Al─O intermediates. These F─Al─O intermediates function as a chemical transition layer between AlF_3_ and NVOPF@C. By integrating with CNTs, they constitute a dense passivation layer that effectively suppresses side reactions and concurrently creates a continuous conductive pathway, which significantly improves the electrical conductivity of the surface coating. The valence state of V is almost unchanged after surface decorating with AlF_3_ (Figure 2f). Two peaks centered at 516.9 eV (V 2p_3/2_) and 524.3 eV (V 2p_1/2_) are consistent with V^4+^ oxidation state spectra, as evidenced by the EPR test results (Figure S8) [22, 23].

Scanning electron microscope (SEM) and Transmission electron microscope (TEM) analyses demonstrate that the spherical morphology of the AF_1_‐NVOPF@C‐2 sample maintained with a diameter of ∼1 µm (Figure 2 g and Figure S2). The interplanar spacing of 0.53 and 0.34 nm, in agreement with that of NVOPF (002) and CNTs (002) crystal planes [24, 25], indicating the successful preparation of samples. Furthermore, a uniform thin AlF_3_ coating of approximately 3 nm is constructed on the surface (Figure 2 h). Nanoscale coatings can enhance interfacial adhesion to substrate materials and shorten the transport paths of ions/electrons. More importantly, the homogeneous coating ensures a consistent electrochemical reaction environment on the material surface, preventing uneven charge transfer caused by thickness variations [26]. TEM‐based energy dispersive X‐ray spectroscopy (EDS) mappings also demonstrate that Al, F, O, C, V, P, and Na are all homogeneously distributed within the particles (Figure 2i and Figure S9).

**FIGURE 3:**
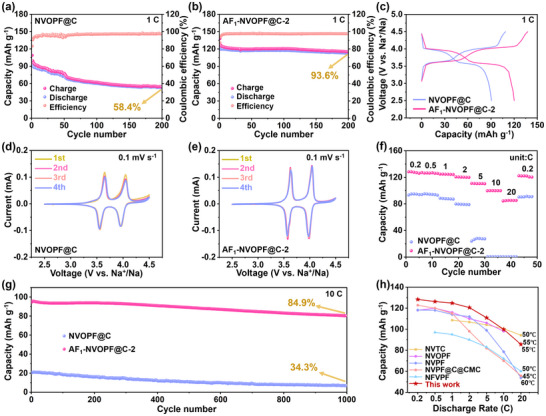
a,b) cycling stability and c) initial charge/discharge profiles at 1 C, d,e) CV curves at 0.1 mV s^−1^, f) rate capability from 0.2 C to 20 C, g) long‐term cycle stability at 10C of NVOPF@C and AF_1_‐NVOPF@C‐2. h) The comparison of rate performance at high temperatures.

First, the effect of AlF_3_ coating on the electrochemical performance of NVOPF@C materials at 25°C and 55°C was evaluated by assembling half cells. NVOPF@C displays 78.3% capacity retention after 100 cycles at 25°C (Figure S10a), and the capacity retention decreases to 58.4% when tested at 55°C (Figure 3a). In comparison, AF_1_‐NVOPF@C‐2 delivers a much higher capacity retention of 92.1% at 25°C (Figure S10b), and this value increases to 93.6% at 55°C (Figure 3b). The Coulombic efficiency of the AlF_3_‐modified cathode is also improved under both ambient and elevated temperatures, especially for the initial cycles. Further, AF_1_‐NVOPF@C‐2 exhibits much longer plateaus and higher capacities than NVOPF@C in the first cycle (Figure 3c). In addition, the voltage decay of AF_1_‐NVOPF@C‐2 is also significantly reduced during cycling (Figure S11). The above results suggest that AlF_3_ coating can significantly suppress undesired structural disruption and parasitic reactions, and the inhibitory effect is more pronounced at high temperatures. The electrochemical behavior of the two cathodes at high temperatures was further examined by cyclic voltammetry (CV). As presented in Figure 3d, e, both NVOPF@C and AF_1_‐NVOPF@C‐2 display two pairs of reversible redox peaks, which correspond well with the main voltage plateaus on charge/discharge profiles (Figure 3c). Two pairs of redox peaks of AF_1_‐NVOPF@C‐2 are much sharper and almost overlap with each other, which implies excellent kinetic behavior and high reversibility. Comparison of CV curves in the initial cycle at 0.1 mV s^−1^ are also provided (Figure S12), the electrode polarization potential difference (ΔE) of the two redox pairs of NVOPF@C and AF_1_‐NVOPF@C‐2 was accurately calculated to be 96 mV/89 mV and 50 mV/61 mV (Table S2), respectively. Obviously, AF_1_‐NVOPF@C‐2 exhibits a smaller ΔE, which implies that the AlF_3_ coating has a positive effect on stabilizing interfacial properties and improving kinetics.

To evaluate the influence of AlF_3_ on power density at high temperature, the rate performance of both cathodes was tested in the range of 0.2 C–20 C (Figure 3f). The AF_1_‐NVOPF@C‐2 cathode exhibits the discharge capacities of 128.3, 126.3, 124.8, 120.6, 110.9, 99.8, and 84.4 mAh g^−1^ at 0.2 C, 0.5 C, 1 C, 2 C, 5 C, 10 C, and 20 C, respectively. In comparison, NVOPF@C delivers the capacities of 93.4, 95.1, 88.5, 79.8, 24, 0.5, and 0.4 mAh g^−1^ at the same currents. When returns to 0.2 C, AF_1_‐NVOPF@C‐2 still shows the capacity of 122.3 mAh g^−1^ with a capacity decay rate of only 4.7%, indicating that the structure maintains even after charging and discharging at high rates of 20 C and excellent reversibility of Na^+^ storage. As the current density increases, the voltage polarization increases (Figure S13). But overall, the voltage polarization of AF_1_‐NVOPF@C‐2 is significantly lower than that of NVOPF@C, and this difference becomes more pronounced at high rates. To distinguish the contribution of AlF_3_‐induced bonding vs. that of intrinsic CNTs conductivity on rate performance, the electrochemical performance of AF_1_‐NVOPF‐2 was evaluated and shown in Figure S14. Notably, its rate capability (Figure S14a) and cycling stability at 1 C (Figure S14b) are inferior to AF_1_‐NVOPF@C‐2 and much better than those of NVOPF@C. This confirms the important role of the AlF_3_ coating and the resulting F─Al─O linkages. Furthermore, the mean discharge voltage (V_m_) of AF_1_‐NVOPF@C‐2 consistently exceeds that of NVOPF@C (Figure S15) from 0.2 C to 20 C, indicating a higher energy density. For comparative purposes, the power and energy densities were determined from the mass of the cathode material (Figure S16). AF_1_‐NVOPF@C‐2 provides a high energy density of 463 Wh kg^−1^ at a low power density of 95 W kg^−1^ and achieves a satisfactory energy density of 283 Wh kg^−1^ as the power density increased to 8966 W kg^−1^. Whereas, NVOPF@C provides a lower energy density of 310 Wh kg^−1^ at a power density of 95 W kg^−1^ and a much lower energy density of 1.3 Wh kg^−1^ when the power density increased to 6780 W kg^−1^. To further elucidate the stabilizing effect of AlF_3_ coating on NVOPF@C under high temperature conditions, charge/discharge tests at a 10 C rate were carried out (Figure 3g). AF_1_‐NVOPF@C‐2 delivers the capacity of 94.6 mAh g^−1^ in the first discharge and maintained at 80.3 mAh g^−1^ over 1000 cycles, corresponding to the capacity retention rate of 84.9%, much higher than NVOPF@C (34.3% capacity retention). Moreover, the high rate cycling stability of AF_1_‐NVOPF@C‐2 at high temperature is superior compared with other reported NVOPF‐based cathode materials for SIBs (Figure 3h and Table S3). This also demonstrates the remarkable function of AlF_3_ as a highly ion‐conductive overlay coating in achieving high power and energy density. In addition, the similar beneficial effects of the AlF_3_ coating were further confirmed in different electrolyte systems, which involve different salts and solvents. As shown in Figure S17, the optimized sample AF_1_‐NVOPF@C‐2 displays negligible differences in capacity at various rates (Figure S17a) and capacity fading at 1 C (Figure S17b) in four electrolytes.

**FIGURE 4:**
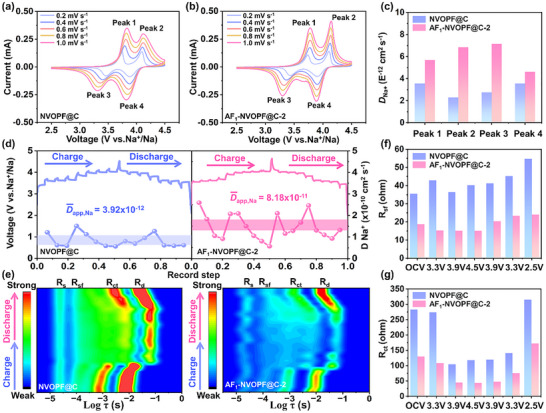
a,b) Cycle voltammetry curves from 0.2 to 1.0 mV s^−1^. c) *D*_Na_ at corresponding redox peaks for the CV method. d) GITT profiles and corresponding Na^+^ diffusion coefficients, e) DRT contour pattern for in‐situ EIS from 2.5 to 4.5 V in the first cycle, f, g) the corresponding statistics for R_sf_ and R_ct_ of NVOPF@C and AF_1_‐NVOPF@C‐2 cathodes.

To assess the effect of AlF_3_ coating on electrochemical reaction kinetics at high temperatures, Na ion diffusion coefficients were calculated using two typical methods. Figure 4a, b illustrates the CV results for the NVOPF@C and AF_1_‐NVOPF@C‐2 cathodes at scan rates of 0.2 to 1.0 mV s^−1^. With scan rate increases, the intensity of the redox peaks is enhanced, accompanied by a shift of the anodic and cathodic peaks to higher and lower potentials, respectively. A linear relationship between the square root of scan rates (*v* ^1/2^) and the peak current (*i*_p_) is established in Figure S18. The diffusion coefficients for Na ion (*D*_CV_) are given by the Randles–Sevcik equation (Equation S1) [27]. The results display that the corresponding *D*_CV_ for peak 1, 2, 3, and 4 in AF_1_‐NVOPF@C‐2 are 5.69 × 10^−12^, 6.85 × 10^−12^, 7.16 × 10^−12,^ and 4.62 × 10^−12^ cm^2^ s^−1^ (Figure 4c), respectively, which are 1.59, 2.99, 2.60, and 1.30 times higher than those of NVOPF@C. Analogously, the GITT test was further employed to evaluate the apparent Na ion diffusion coefficient (*D*_app_, _Na_) over a complete charge/discharge cycle at 0.5 C (Figure 4d). The single titration curves and the details are presented in Figure S19 and Equation S2 [28]. The calculated Na^+^ diffusion coefficients of AF_1_‐NVOPF@C‐2 throughout the entire charge‐discharge process are about 8.18 × 10^−11^ cm^2^ s^−1^, which is significantly higher than that of NVOPF@C (about 3.92 × 10^−12^ cm^2^ s^−1^). It indicates that AF_1_‐NVOPF@C‐2 has a higher ionic conductivity and faster Na ion diffusion kinetics. Additionally, HRTEM observations (Figure S20) reveal that the (200) and (002) crystal‐plane spacings reversibly contract and expand during charge/discharge cycles in response to sodium‐ion extraction and insertion, confirming the structural reversibility. The corresponding schematic model of the microstructural evolution is shown in Figure S21 [29].

For a comprehensive elucidation of the cathode interface dynamic evolution mechanism, in ‐situ electrochemical alternating current electrochemical impedance spectroscopy (EIS) technique was employed to monitor the interfacial impedance changes at different states during the charging and discharging processes (Figure S22a). During the first cycle, the impedance values of both cathodes exhibit a pattern of gradual decrease during charging followed by an increase during discharging. The difference is that AF_1_‐NVOPF@C‐2 consistently exhibits smaller charge transfer resistance (R_ct_) values than NVOPF@C, which suggests that the reaction kinetics are accelerated due to the AlF_3_ coating acting as an ion transport accelerator, thereby improving the Na ion transport efficiency. The EIS spectra were transformed into a function of frequency by distribution of relaxation time (DRT) analysis, which provides a more intuitive indication of the resistance of the different portions through the integrated area of specific peaks (Figure 4e and Figure S22b,c). The peaks at different time scale partitions are indicative of their respective resistances [30]. Where the peaks at about 10^−5^ s time scale represent the contact resistance (R_s_) between the cathode particles, the peaks at 10^−4^ s reflect the surface film resistance (R_sf_), the peaks between 10^−4^ and 10^−2^ s indicate the R_ct_, which coincides with the frequency range of the semi‐cycle in the impedance spectra, and the peaks at 10^−2^ s represent the ion diffusion resistance (R_d_) in the electrodes. The R_sf_ and R_ct_ values for NVOPF@C are greatly higher than those of AF_1_‐NVOPF@C‐2 throughout the GCD test. The R_sf_ values of AF_1_‐NVOPF@C‐2 remain relatively stable with the change of charging/discharging state (Figure 4f), indicating that AF_1_‐NVOPF@C‐2 obtains a more stable CEI layer due to the presence of the AlF_3_ coating. Regarding the R_ct_ part (Figure 4g), the trend of the DRT spectroscopy resembles that of the half arc in Figure S22a. The charging process shows a gradual decrease in the impedance value with increasing voltage, which is resulted from the low Na ion extraction barrier in the Na ion poor phase at high voltages [31], and the R_ct_ for AF_1_‐NVOPF@C‐2 further decreases from 129.6 to 43.9 Ω. During the discharging process, R_ct_ and its peak position undergo the opposite process, and the impedance value further increases to 172.5 Ω when the voltage drops to 2.5 V. The increase in resistance is attributed to tardive Na ion mobility in the Na ion rich phase [32]. Overall, reversible DRT spectroscopy reveals the formation of a stable CEI interface and a significantly enhanced ion diffusion rate during charging/discharging of AF_1_‐NVOPF@C‐2.

The charge compensation mechanism of AF_1_‐NVOPF@C‐2 is elucidated via ex‐situ XPS (Figure S23). For a pristine electrode, the peaks of V 2p_1/2_ and V 2p_3/2_ correspond to the tetravalent state of vanadium. Upon charging to 4.5 V, both peaks shift toward higher binding energies, to 524.8 and 517.3 eV, respectively, suggesting the conversion of V^4+^ to V^5+^ during the deintercalation of the two Na ions. After complete discharge to 2.5 V, the V 2p_1/2_ (524.3 eV) and V 2p_3/2_ (517.0 eV) peaks return to their original states, demonstrating that V^5+^ reduces to V^4+^, along with the intercalation of two Na ions. These results confirm that the Na ion storage capacity of AF_1_‐NVOPF@C‐2 is attributed to the V^4+^/V^5+^ redox pair, demonstrating the high degree of valence reversibility [33]. To further investigate the reason for the enhanced cycling stability and kinetics after AlF_3_ decoration, the surface morphology of both electrodes after 1000 cycles at 10 C at 55°C was investigated by SEM (Figure S24). The NVOPF@C cathode exhibits severe cracking and irregular expansion after cycling, resulting in a hollow structure, attributed to the uneven stress release and structural degradation induced by high temperature electrochemical cycling [34]. In contrast, AF_1_‐NVOPF@C‐2 displays only minor pores and superficial cracks. Cross‐sectional analysis further reveals negligible volumetric change and a preserved flat surface for AF_1_‐NVOPF@C‐2, whereas the uncoated electrode undergoes significant volume expansion. This indicates that the AF_1_‐NVOPF@C‐2 is well protected by AlF_3_ coating, which significantly reduces the side reactions under high temperature reaction and maintains the structural stability with low volume change.

**FIGURE 5:**
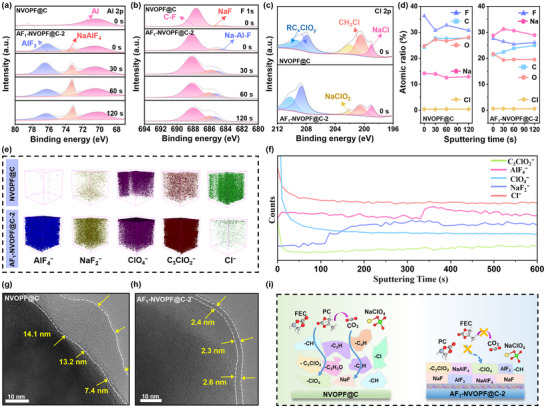
Interfacial chemistry of the NVOPF@C and AF_1_‐NVOPF@C‐2 cathodes: XPS of the CEI: a) Al 2p and b) F 1s during Ar_n_^+^ sputtering, c) Cl 2p and d) quantified atomic ratios of the elements with the increasing sputtering time. e) TOF‐SIMS 3D‐mapping images of several representative secondary ion fragments obtained from the cathodes after cycling. f) depth profiles of several representative secondary ion fragments obtained from the cathodes based on corresponding TOF‐SIMS results. g, h) HRTEM images of CEI layer cycling at 55°C for 100 cycles. i) Mechanism diagram of CEI formation.

To further understand the optimization effect of AlF_3_ coating on CEI, the components of the CEI layer were investigated by XPS measurements. The results revealed that the CEI on the AF_1_‐NVOPF@C‐2 surface shows additional peaks (Figure 5a), which are attributed to substances such as AlF_3_ (76.2 eV) and NaAlF_4_ (73.1 eV) [35]. The formation of NaAlF_4_ may be induced by FEC additive decomposition, which creates a F‐rich environment that promotes partial dissolution of the AlF_3_ coating. This process releases Al^3+^, enabling its combination with Na^+^ from the electrolyte [36, 37]. The generated NaAlF_4_ layer acts as an ionic conductor and embedded in the CEI layer. Together with the undissolved AlF_3_ coating layer, making Na^+^ easy to transport across the CEI and at the same time suppresses the side reactions and enhances the interfacial stability [38]. As sputtering time increases, the NaAlF_4_ content gradually rises due to the accelerated oxidative decomposition of FEC during the reaction, which provides more fluorine sources and drives the reaction to proceed further. Moreover, AlF_3_ provides additional F^−^, which alters the chemical environment of the reaction system and promotes the synergistic decomposition of FEC and NaClO_4_, leading to the formation of NaF [39]. This is also demonstrated in the F 1s spectrum. As shown in Figure 5b, two peaks associated with C−F (688.1 eV, from PVDF) [40] and NaF (685.7 eV) [41] can be clearly observed in the CEI of AF_1_‐NVOPF@C‐2, as well as an additional peak related to the Na−Al−F (685.1 eV) [42] species. The additional Na−Al−F peaks further indicate the formation of NaAlF_4_ species. At the same time, the higher content of NaF detected in the CEI of the AF_1_‐NVOPF@C‐2 cathode further confirms the significant role of AlF_3_ in promoting the synergistic decomposition reaction between FEC and NaClO_4_. Since NaF has a low Na^+^ diffusion energy barrier (0.12 eV) and a high Young's modulus (31.4 GPa), the NaF‐rich CEI layer facilitates Na^+^ diffusion and mitigates particle cracking [43]. Furthermore, minor amounts of substances originating from the NaClO_4_ electrolyte, such as RC*_x_* ClO*_y_*, NaClO_2_, CH_3_Cl, and NaCl, could be detected on the cathode surface (Figure 5c). The difference is that the surface of AF_1_‐NVOPF@C‐2 shows a higher content of chlorinated organic compounds (e.g., RC*_x_* ClO*_y_*) and ClO_4_^−^, while a lower content of inorganic substances (e.g., NaClO_2_ and NaCl). These chlorine‐containing inorganic substances are considered to be undesirable products that impede the diffusion of Na ions [44]. This reflects the fact that the AlF_3_‐coating inhibits the side reaction of direct reduction from ClO_4_^−^ to inorganic Cl^−^ and NaClO_2_, and instead stabilizes the chemical state of Cl by enhancing the synergistic interaction between FEC and ClO_4_^−^ to form an inorganic‐dominated composite. Further depth profiling of CEI was conducted by Ar_n_^+^ cluster sputtering to reveal the elemental distribution (Figure 5d). For AF_1_‐NVOPF@C‐2, lower C and O fractions and higher Na fractions are shown for all sputtering depths attributed to the presence of the AlF_3_ coating. Thus, the contact between electrolyte and active material can be effectively blocked to reduce the oxidative decomposition of solvent molecules while promoting the generation of the inorganic NaF‐rich interfacial layer.

The time‐of‐flight secondary ion mass spectrometry (TOF‐SIMS) was employed to quantify the interfacial composition and visualize the spatial distribution of CEI chemical species. The 3D spatial distribution plots are displayed in Figure 5e and Figure S25a. Among them, the inorganic sputtered fragments of AlF_4_^−^ (standing for AlF_3_ and NaAlF_4_) and NaF_2_^−^ (standing for NaF) are formed by AlF_3_ coating layer‐electrolyte interactions as well as electrolyte decomposition. Chlorine‐containing species originate from both organochlorine compounds and inorganic chlorine fragments generated by the decomposition of NaClO_4_. The other organic fragments are derived from the oxidative decomposition of the solvent molecules (PC) with the FEC. In conjunction with the intensity depth distribution of different secondary ion fragments during the 600 s sputtering time (Figure 5f and Figure S25b‐d), the CEI on the surface of AF_1_‐NVOPF@C‐2 cathode exhibits a typical mosaic‐like component distribution [45]. The surface layer is primarily composed of a minor amount of organic carbon species and organochlorine fragments generated from electrolyte decomposition, whereas the inner layer comprises an interwoven ternary fluoride composite of NaF/AlF_3_/NaAlF_4_. The HRTEM image shows that the thickness of CEI on NVOPF@C cathode is 7–15 nm (Figure 5 g). In comparison, a CEI layer of ∼3 nm is observed in AF_1_‐NVOPF@C‐2 cathode (Figure 5 h). Even after 500 and 1000 cycles, NVOPF@C still exhibits a non‐uniform CEI layer (Figure S26a,b), whereas AF_1_‐NVOPF@C‐2 maintains a uniformly thin CEI layer of about 3 nm (Figure S26c,d). This clearly indicate**s** the durability of the CEI layer after AlF_3_ coating. On this basis, Figure 5i shows a diagram of the distribution of CEI on both cathodes. All the results demonstrate that AlF_3_ is conducive to the reconstruction of a uniform and dense CEI layer, which facilitates Na^+^ transport, reduces interfacial impedance, and enhances high temperature performance.

**FIGURE 6:**
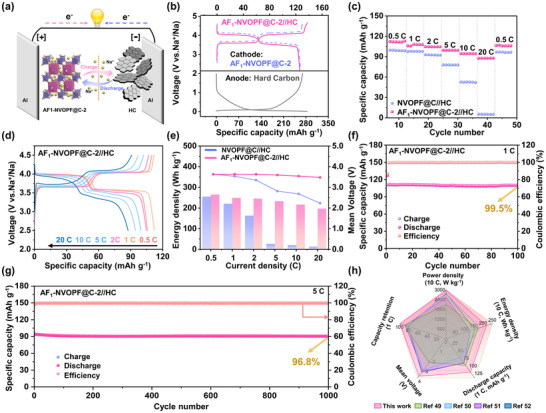
a) Schematic diagram of full cell operation principle. b) GCD profiles of the AF_1_‐NVOPF@C‐2//HC full cell, the AF_1_‐NVOPF@C‐2 electrode, and the contrastive HC electrodes in half cells. c) Rate performance and d) Corresponding GCD profiles, e) energy density and Vm of AF_1_‐NVOPF@C‐2//HC and NVOPF@C//HC. Cycling performances at f) 1C and g) 5C of AF_1_‐NVOPF@C‐2//HC. h) Radar chart for performance comparison.

The practical viability of AF_1_‐NVOPF@C‐2 as a cathode material for SIBs was assessed by assembling full cells with commercially available hard carbon (HC) anodes (Figure S27). To enhance the initial coulombic efficiency and cycling stability of the HC, pre‐sodiation was conducted at various states to mitigate adverse effects [46, 47]. As shown in Figure S28, the 100% pre‐sodiated HC anode exhibits the best electrochemical performance, therefore, it was selected as the anode for the full cell assembly. The working principle is shown in Figure 6a, which indicates the reversible insertion/extraction of Na ions between cathode and anode [48]. Figure 6b shows the charge/discharge profiles of half cells (AF_1_‐NVOPF@C‐2//Na and HC//Na) and full cells (AF_1_‐NVOPF@C‐2//HC) at 0.2 C. The assembled AF_1_‐NVOPF@C‐2//HC full cell exhibits slightly lower voltage plateaus and a capacity decrease (1.7%) when compared with the AF_1_‐NVOPF@C‐2//Na half‐cell. The rate capability was further tested to evaluate the power density (Figure 6c). The AF_1_‐NVOPF@C‐2//HC full cell exhibits an average discharge capacity of 112.3 mAh g^−1^ at 0.5 C (based on the cathode, 1C = 130 mAh g^−1^) and maintains a high specific capacity of 87.6 mAh g^−1^ at 20 C, which are much higher than that of NVOPF@C//HC (the capacity almost disappears at 20 C). With the current density restored to 0.5 C, a reversible capacity of 106.4 mAh g^−1^ is achieved. Figure 6d demonstrates the charge/discharge profiles of AF_1_‐NVOPF@C‐2//HC cell at different rates. The charge/discharge specific capacity gradually decreases, and polarization slightly increases with the increase of currents, but the charge/discharge plateau remains clearly visible even at 20 C. The energy density and power density of the full cell were calculated using the total mass of active materials in both electrodes, with the results presented in Figure 6e and Figure S29. Compared with NVOPF@C//HC, AF_1_‐NVOPF@C‐2//HC shows a higher average operating voltage (V_m_) and maintains 74.3% of its energy density (197 Wh kg^−1^) even when the power density increases to 5869 W kg^−1^, revealing great potential for practical applications. The long cycle stability of AF_1_‐NVOPF@C‐2//HC full cell was also evaluated. The capacity retention is 99.5% after 100 cycles at 1 C, showing almost negligible capacity degradation (Figure 6f). More impressively, it can still provide a discharge capacity of 96.5 mAh g^−1^ after 1000 cycles at 5 C, corresponding to a high capacity retention of 96.8% and an extremely low capacity degradation rate of 0.0032% per cycle (Figure 6g). Compared with various other reported full batteries (Figure 6h), the excellent performance of our assembled AF_1_‐NVOPF@C‐2//HC is in an advantageous position and shows great potential for commercial applications [49, 50, 51, 52].

## 3 Conclusion

In conclusion, we present a facile strategy to engineer an ultrathin AlF_3_ coating (about 3 nm) on NVOPF@C cathodes, which significantly improves the high rate cycling stability, especially at 55°C. This approach not only addresses the critical challenge of interfacial instability in NVOPF cathodes under high temperature conditions but also establishes an efficient electron conducting framework. Systematical investigations reveal that AlF_3_ interacts with NaClO_4_ and FEC during cycling, facilitating the in‐situ construction of a stable CEI. The resulting inorganic‐dominated composite CEI is enriched with a ternary fluoride mixture of NaF/AlF_3_/NaAlF_4_, exhibiting uniform, dense, and robust properties. The newly constructed CEI not only promotes Na ion mobility and rate capability for the pristine cathode but also substantially improves the interfacial stability and extends cycle life. When assembled with HC anodes, the AF_1_‐NVOPF@C‐2//HC full cell delivers exceptional long‐term stability (96.8% capacity retention at 5 C after 1000 cycles), along with high energy and power densities, highlighting its potential for next‐generation SIBs. This work highlights the significant importance of uniform coating design and in‐situ formation of durable interfaces in achieving superior high rate cycling capability at elevated temperature.

## 4 Experimental Section

### 4.1 Materials preparation

#### 4.1.1 Synthesis of Na_3_V_2_(PO_4_)_2_O_2_F@CNT (NVOPF@C)

At first, 4 mmol NH_4_VO_3_ (AR, Aladdin) and 2 mL H_2_O_2_ (30%, purchased from Chengdu Kelong Chemical Co., Ltd.) were added into 10 mL deionized (DI) water and stirred for 1 h. A clear orange transparent solution was obtained and recorded as solution A. Meanwhile, 80 mg carbon nanotubes (CNTs, purchased from XFNANO) were added into 10 mL DI water and sonicated for 1 h to disperse homogeneously, which was recorded as solution B. After that, solutions A and B were mixed well and stirred continuously for 30 min. Second, 4 mmol NaH_2_PO_4_·2H_2_O (AR, Aladdin), 2 mmol NaF (AR, Sinopharm) were poured sequentially into the above solution with stirring for 30 min at room temperature. Finally, 60 mL PEG400 was added and the mixture was stirred for 1 h. The above product was transferred into a 100 mL Teflon‐lined autoclave and kept at 180°C for 24 h. When cooling down, the obtained precipitate was washed three times with DI water and alcohol, and then dried at 80°C for 24 h.

#### 4.1.2 Synthesis of AlF_3_‐Modified Na_3_V_2_(PO_4_)_2_O_2_F@CNT

(AF*_x_*‐NVOPF@C‐*y*, where *x* represents the mass fraction of AlF_3_ coating layer, which is 0.5, 1, 2, 5, and 10 wt%, respectively; *y* is marked as 1, 2, and 3, which represent the pH value of 1, 7, and 11 for the adjusted coating solution, respectively. First, 200 mg of NVOPF@C was dispersed in 20 mL of DI water. Subsequently, 8.93 mg Al(NO_3_)_3_·9H_2_O (AR, Macklin) was slowly added into the mixture. A separate homogeneous aqueous solution was prepared by dissolving 2.65 mg NH_4_F (AR, Aladdin) in 20 mL of DI water, which was then added dropwise to the above solution. Finally, the pH of the resulting solution was regulated to 1, 7, and 11 using HF or NH_3_·H_2_O, respectively. After the evaporation of water by continuous stirring at 80°C for 5 h, it was transferred to a vacuum drying oven and the obtained powder was calcined at 400°C for 5 h in Ar. The stoichiometric molar ratio of Al and F is 1:3, and the amount of AlF_3_ coating is 1 wt%. Samples with 0.5, 2, 5 and 10 wt % AlF_3_ coating were synthesized under otherwise identical conditions. Pure AlF_3_ originated from Al(NO_3_)_3_·9H_2_O and NH_4_F solution without NVOPF and CNTs was also prepared. An additional control sample of AlF_3_‐coated NVOPF without CNTs was also synthesized under optimized experimental conditions (pH = 7, 1 wt% of AlF_3_ coating), labeled AF_1_‐NVOPF‐2.

### 4.2 Materials Characterization

The XRD equipped with Cu‐Kα radiation (λ = 1.5406 Å) was utilized to investigate the crystalline information. The morphology of the materials was investigated by SEM (JSM‐7500F, JEOL) and TEM (FEI Tecnai G2 F30), providing a detailed exploration of the microstructure. Raman spectroscopy (LabRAM HR Evolution&SmartSPM, HORIBA, Japan) was recorded to characterize the structural information. The chemical composition and valence states were comprehensively assessed using XPS (AXIS SUPRA+, Kratos, Japan). TOF‐SIMS (nano TOFIII, Ulvac‐Phi, Japan) was employed to evaluate the composition properties of CEI. The carbon content was determined via a thermogravimetric analyzer (TGA, NETZSCH STA449F5). The elemental content of the samples was analyzed by ICP‐OES (Thermo ICAP7000).

### 4.3 Electrochemical Measurements

The electrochemical performances of all samples were measured with standard CR2016 coin cells. Both the cathode and the HC anode were composed of active material, acetylene black, and polyvinylidene fluoride with a weight ratio of 7:2:1. The prepared paste was uniformly coated on the surface of Al foil, dried for 12 h, and cut into discs with a diameter of 10 mm. In the half cells, the loading of the cathode active material was about 1.0–1.5 mg cm^−2^, with sodium tabs as the anode electrode. HC was pretreated by galvanostatic charge/discharge in half cells and discharged to 50% (D50), 100% (D100) of their total capacity, and HC discharged then charged until reaching 50% of their total capacity (C50). Then, the Na‐ion full cells were assembled with all kinds of hard carbon and the AF_1_‐NVOPF@C‐2. In the full cells, the loading of the HC anode needs to be slightly higher than that of the AF*_x_*‐NVOPF@C‐*y* cathode to ensure the complete reaction. The mass ratio of the anode material to the cathode material was maintained at about 1:2. Both half cells and full cells were assembled in an argon‐filled glove box with glass fiber as the separator and 1 M NaClO_4_ in PC with 5% fluoroethylene carbonate as the electrolyte. The electrochemical performance and GITT were tested with the LAND battery test system (CT3001A). Furthermore, EIS and CV tests were conducted by an electrochemical workstation (Admiral prime potentiostat) to comprehensively evaluate the electrochemical behavior. The electrochemical performance tests were performed within a voltage window of 2.5 to 4.5 V.

## Conflicts of Interest

The authors declare no conflicts of interest.

## Supporting information




**Supporting File**: advs73812‐sup‐0001‐SuppMat.docx.

## Data Availability

The data that support the findings of this study are available from the corresponding author upon reasonable request.
