# Supplementary material for: AlF3 Mediated In‐Situ Cathode Interface Stabilization Enables High‐Rate and Long‐Life Na‐Ion Batteries at Elevated Temperature
Source: Adv Sci (Weinh). 2026 Jan 14;13(10):e22907. doi: 10.1002/advs.202522907 (PMC12915226; doi:10.1002/advs.202522907)
Supplement: Supplementary file 1 — Supporting File: advs73812‐sup‐0001‐SuppMat.docx. [file ADVS-13-e22907-s001.docx]

**Supporting Information**

AlF_3_ Mediated In-Situ Cathode Interface Stabilization Enables High-Rate and Long-Life Na-Ion Batteries at Elevated Temperature

Ya-Meng Yin, Qinxia Liu, Zhiyuan Zhang, Weixiao Wang, Cunyuan Pei,* Fangyu Xiong, Jinghui Chen, Qinyou An,* Cai-Hong Yang, and Dong-Sheng Li*

Y. M. Yin, Q. Liu, Z. Zhang, C. Pei, C. H. Yang, D. S. Li

Key Laboratory of Inorganic Nonmetallic Crystalline and Energy Conversion Materials, College of Materials and Chemical Engineering, China Three Gorges University, Yichang, Hubei, 443002, China

E-mail: [peicunyuan@ctgu.edu.cn](mailto:peicunyuan@ctgu.edu.cn), [lidongsheng1@126.com](mailto:lidongsheng1@126.com)

Y. M. Yin, C. Pei, C. H. Yang, D. S. Li

Hubei Three Gorges Laboratory, Yichang, Hubei, 443007, China

W. Wang, J. Chen, Q. An

State Key Laboratory of Advanced Technology for Materials Synthesis and Processing, Wuhan University of Technology, Wuhan 430070, China

E-mail: [anqinyou86@whut.edu.cn](mailto:anqinyou86@whut.edu.cn)

F. Xiong

National Engineering Research Center for Magnesium Alloys, College of Materials Science and Engineering, Chongqing University, Chongqing 400044, China


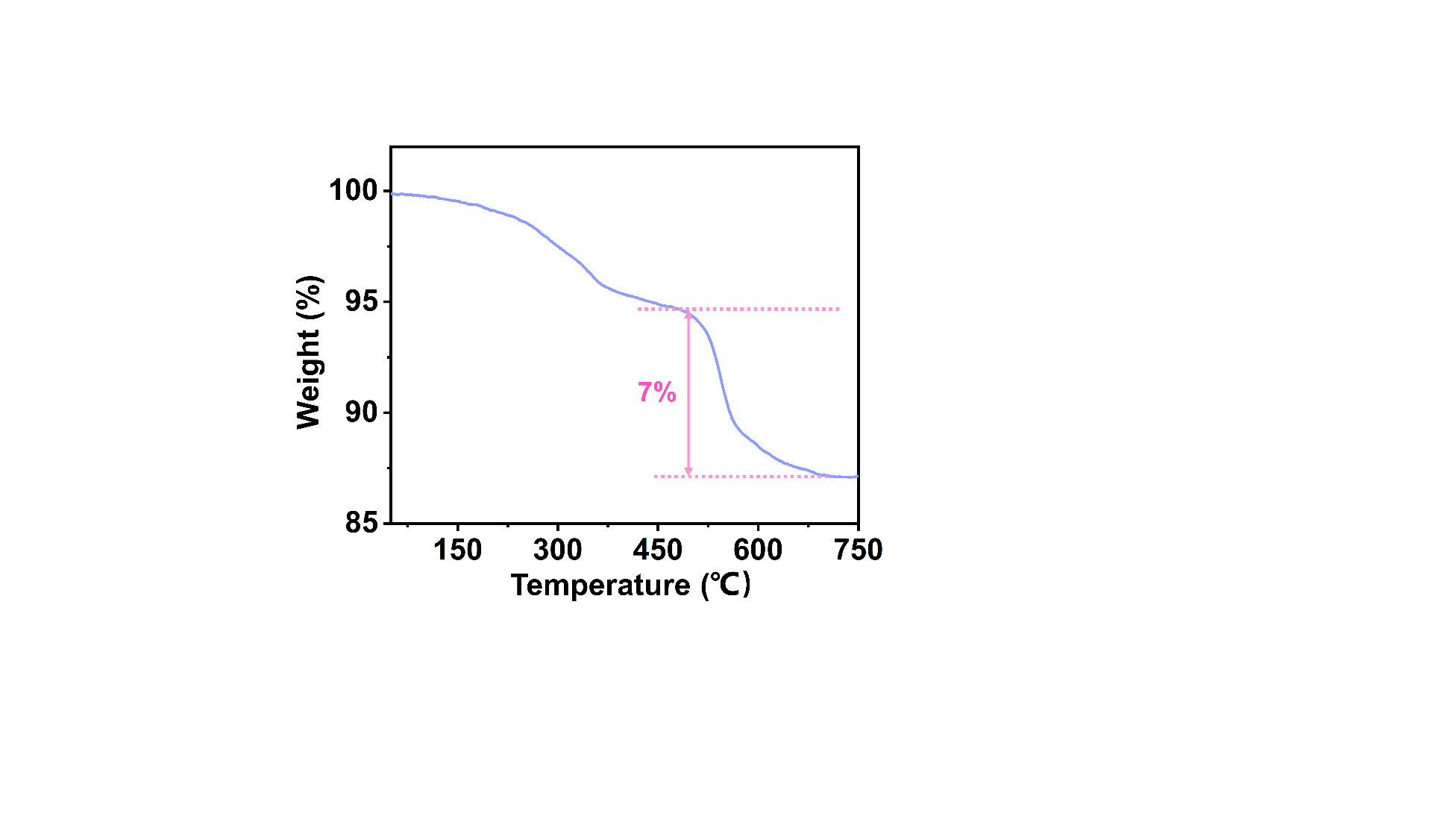


**Figure S1.** Thermogravimetric analysis curve of NVOPF@C.


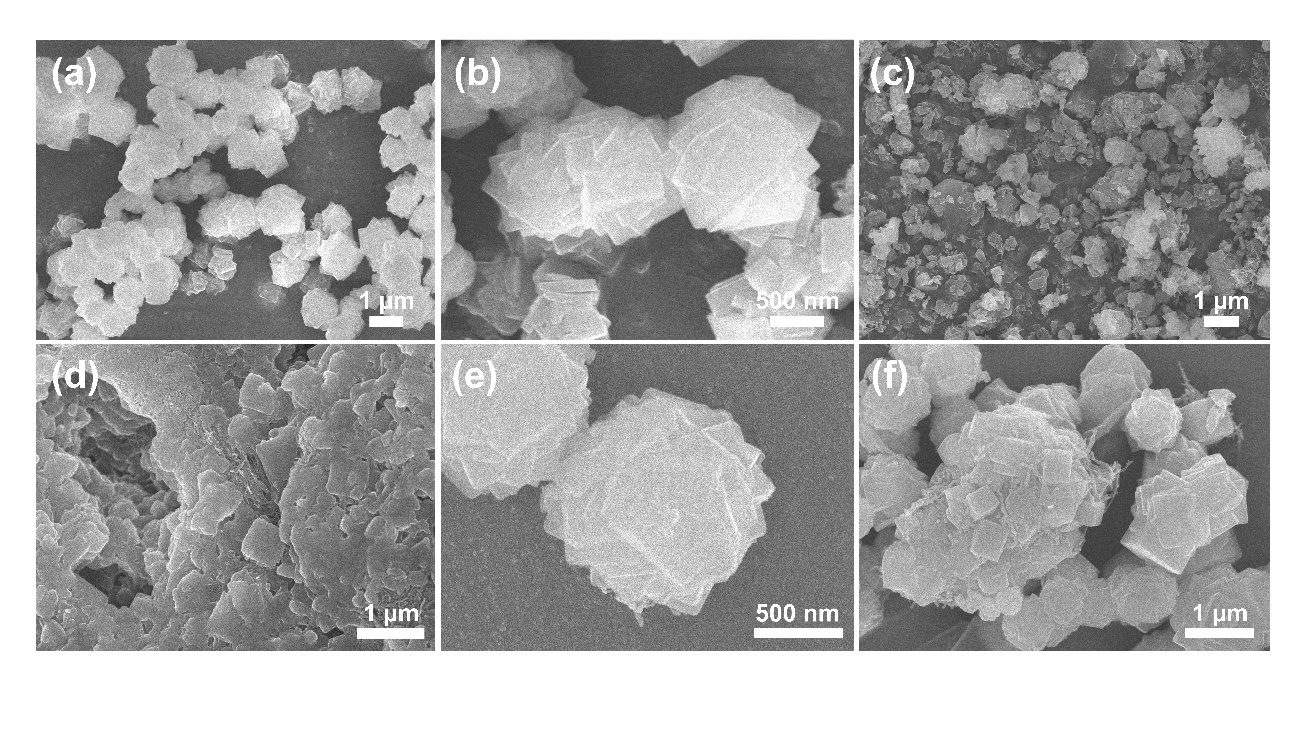


**Figure S2.** Magnified SEM images of a, b) NVOPF@C, c) Sample of 1% AlF_3_ coating without pH adjustment (pH = 4), d) AF_1_-NVOPF@C-1, e) AF_1_-NVOPF@C-2 and f) AF_1_-NVOPF@C-3.


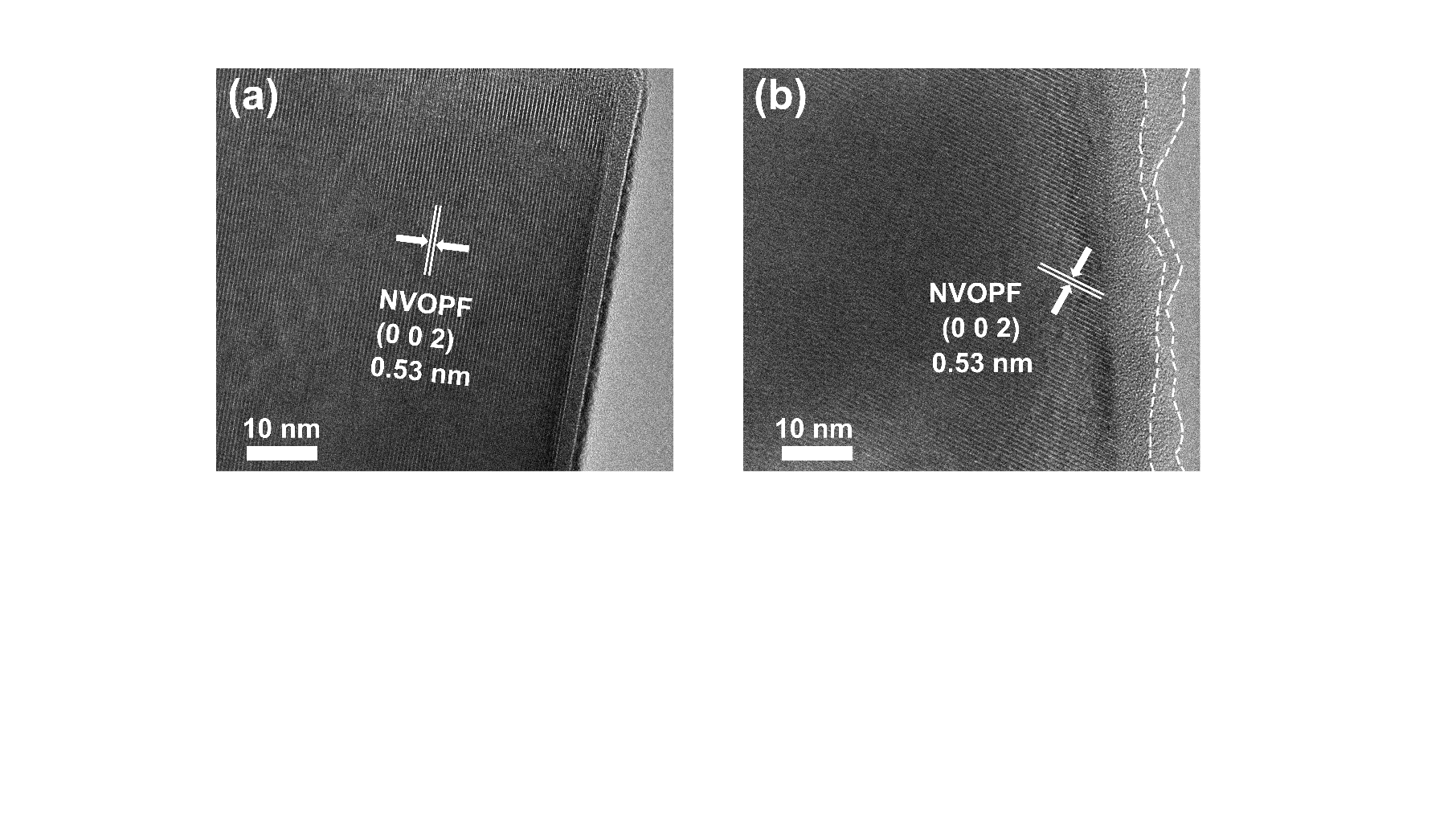


**Figure S3.** HRTEM images of a) AF_1_-NVOPF@C-1 and b) AF_1_-NVOPF@C-3.

As shown in Figure S2a, b, the uncoated NVOPF@CNT samples exhibit a spherical morphology consisting of nanocubes. When synthesizing AlF_3_-modified NVOPF@C, the initial solution without pH adjustment has a pH of 4. The resulting sample (Figure S2c) fails to retain the NVOPF@C morphology and breaks into fragments. After adjusting the pH to 1 with HF, the sample AF_1_-NVOPF@C-1 displays severe aggregation (Figure S2d) and does not form a uniform AlF_3_ coating (Figure S3a). This can be attributed to the highly acidic condition, which may increase the solubility of AlF_3_ and affect its nucleation and growth. Similarly, when the pH is adjusted to 11 using NH_3_·H_2_O, the sample AF_1_-NVOPF@C-3 also shows pronounced aggregation (Figure S2f) and non-uniform AlF_3_ coating (Figure S3b). A highly alkaline environment may promote the formation of mixed hydroxides, thereby affecting the uniformity of the AlF_3_ coating. Only at a pH of 7, adjusted with NH_3_·H_2_O, can the original spherical morphology of NVOPF@CNT and a uniform AlF_3_ coating be achieved (Figure S2e).


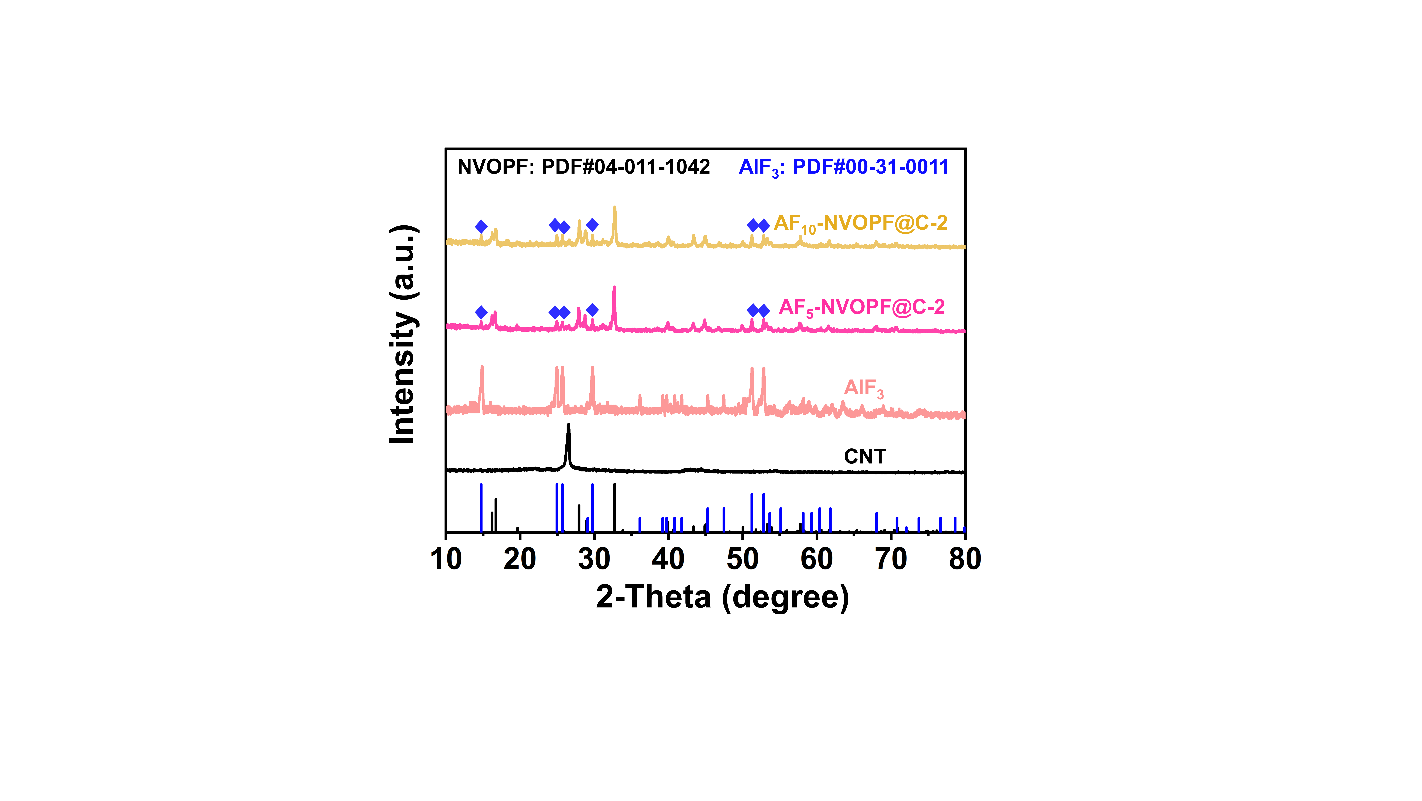


**Figure S4.** XRD patterns of AlF_3_, AF_5_-NVOPF@C-2 and AF_10_-NVOPF@C-2.

**
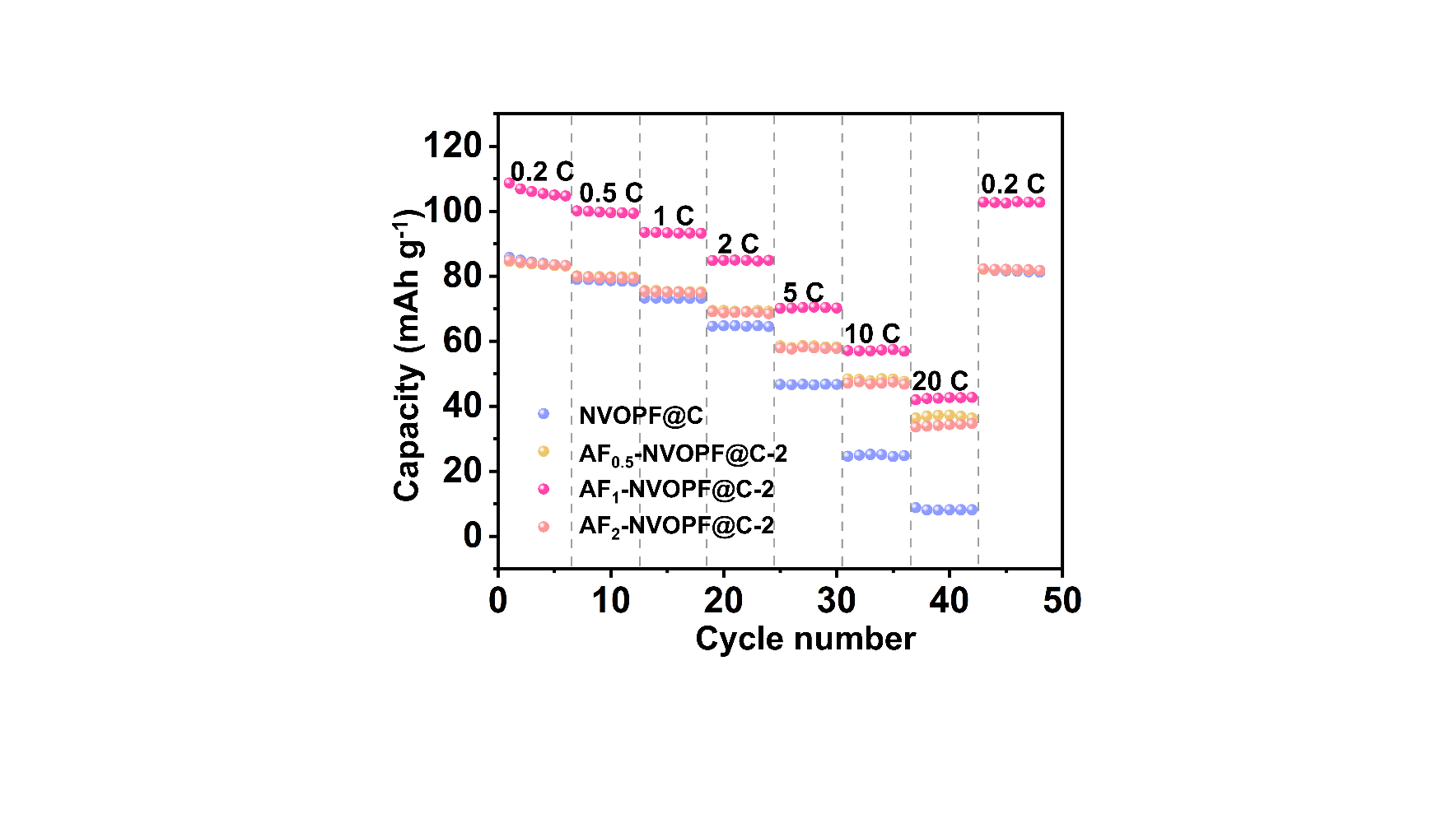
**

**Figure S5.** Rate performance of NVOPF@C, AF_0.5_-NVOPF@C-2, AF_1_-NVOPF@C-2 and AF_2_-NVOPF@C-2 at 25 °C.


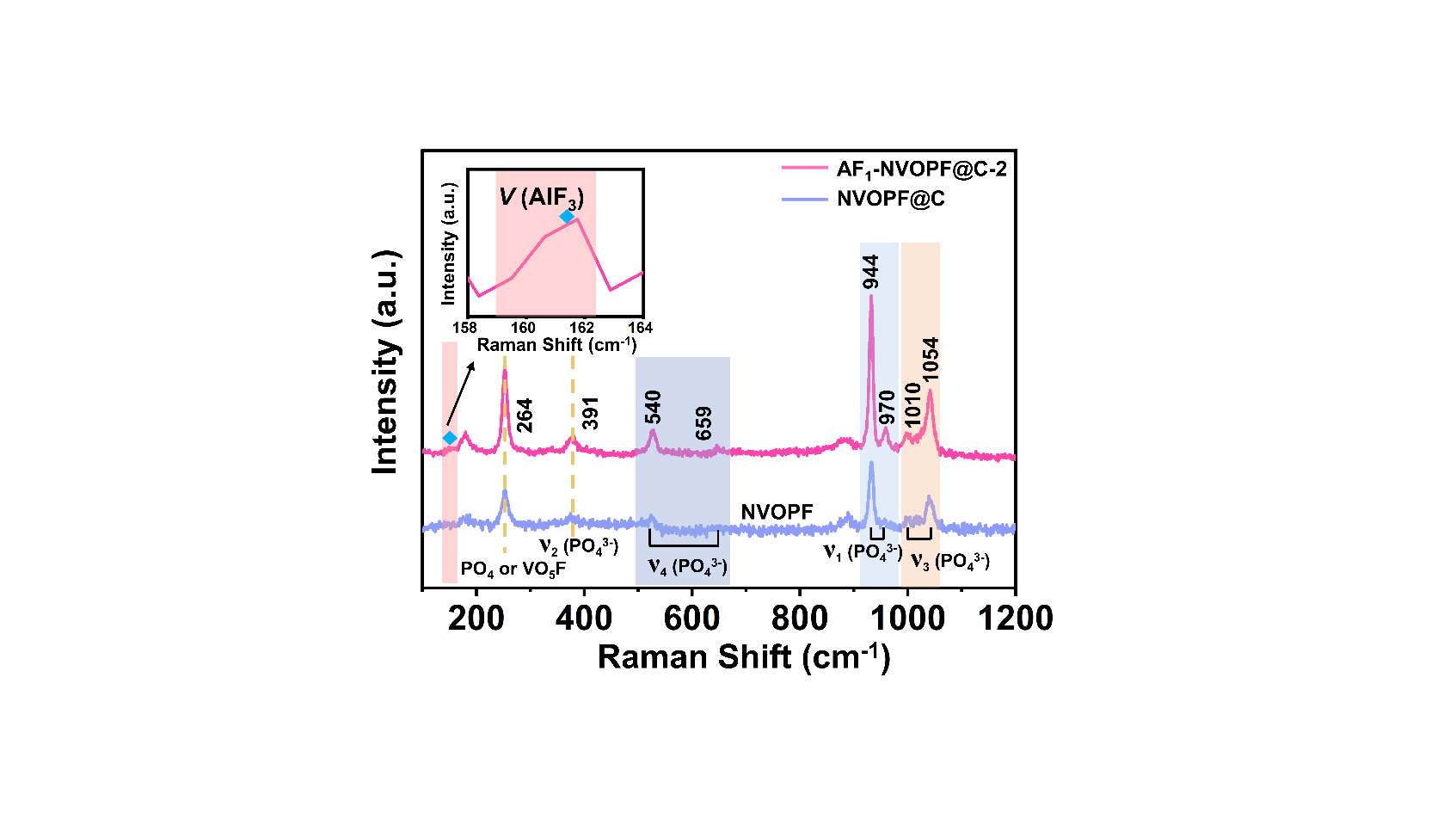


**Figure S6.** Raman spectra of NVOPF@C and AF_1_-NVOPF@C-2.

Raman spectroscopy can generally provide the detailed information of the surface chemical bonding and the structure of samples. A prominent peak at 264 cm^−1^ is observed in Figure S5, corresponding to the vibrational modes of the [PO_4_] tetrahedron or the [VO_5_F] octahedron. Additionally, a peak at 391 cm^−1^ is attributed to the ν_2_ (E) modes of PO_4_^3−^, while bands in the range of 537 to 663 cm^−1^ are associated with ν_4_ vibrations. It is noteworthy that the strong and sharp peaks at 944 cm^‒1^ and 970 cm^−1^ represent the symmetric stretching vibrations of ν_1_ (PO_4_^3−^), whereas the peaks at 1010 cm^‒1^ and 1054 cm^−1^ belong to the ν_3_ mode stretching vibrations of the P−O bond.^[1]^


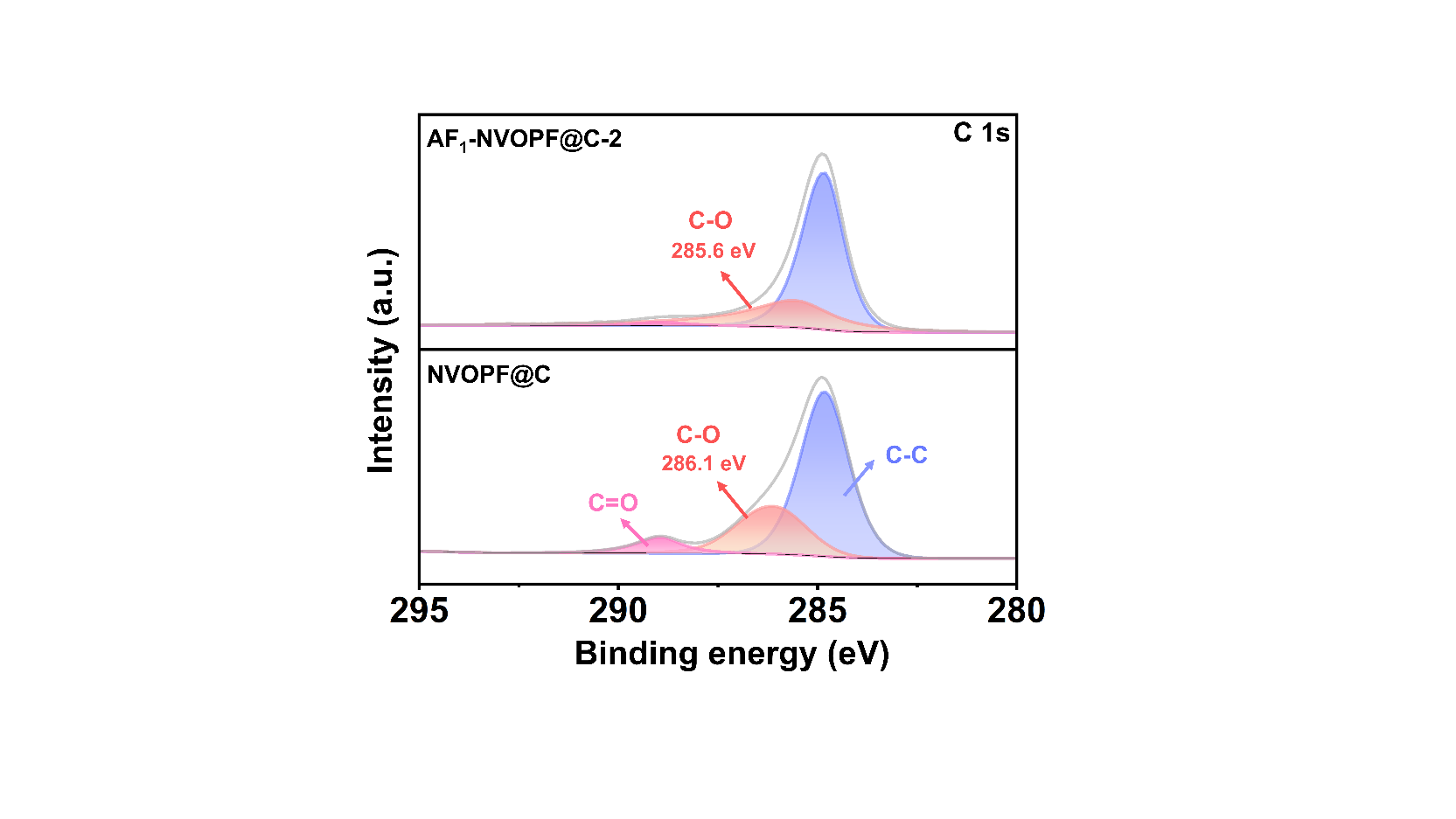


**Figure S7.** C 1s XPS spectra of NVOPF@C and AF_1_-NVOPF@C-2

**
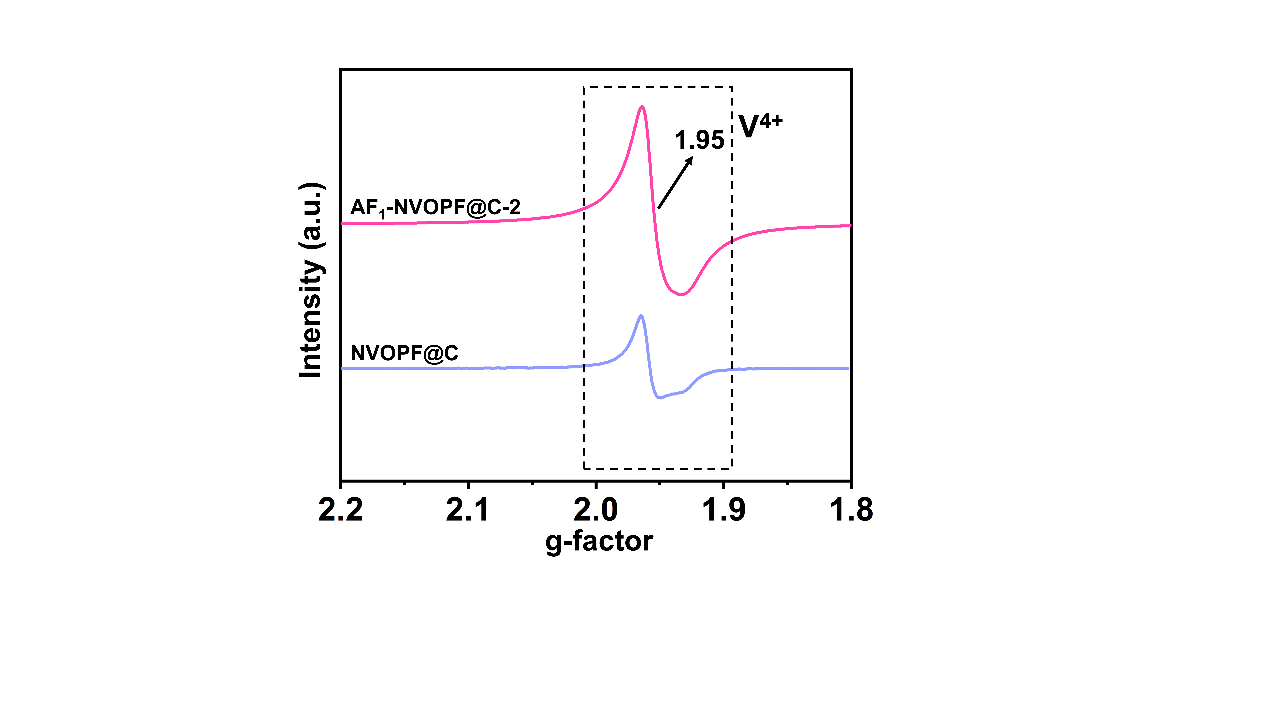
**

**Figure S8.** EPR results of NVOPF@C and AF_1_-NVOPF@C-2.

**
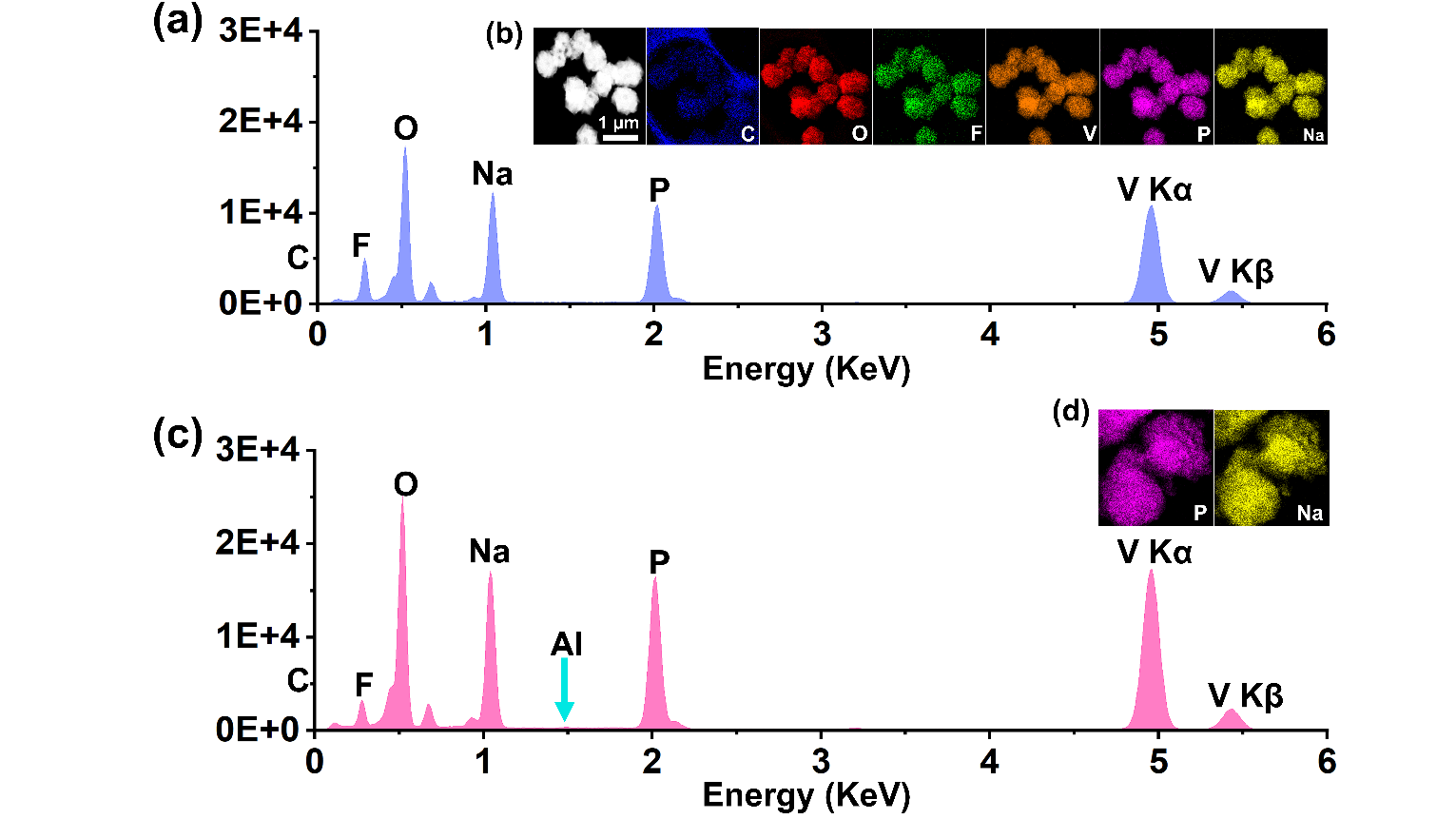
**

**Figure S9.** a, c) EDS results, b, d) mapping images of NVOPF@C and AF_1_-NVOPF@C-2.


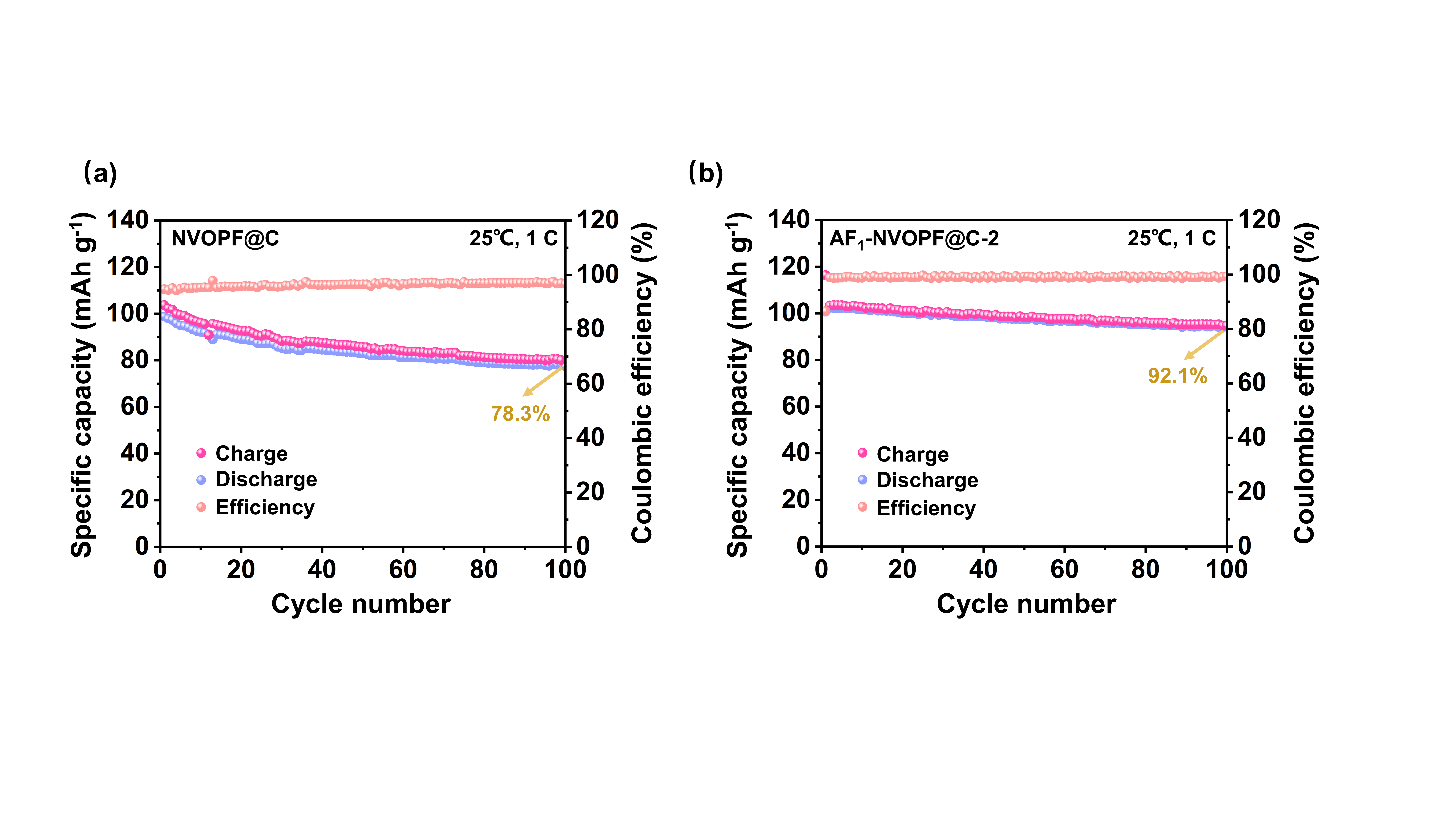


**Figure S10.** Cycling performance of a) NVOPF@C and b) AF_1_-NVOPF@C-2 at 1 C at 25 °C.
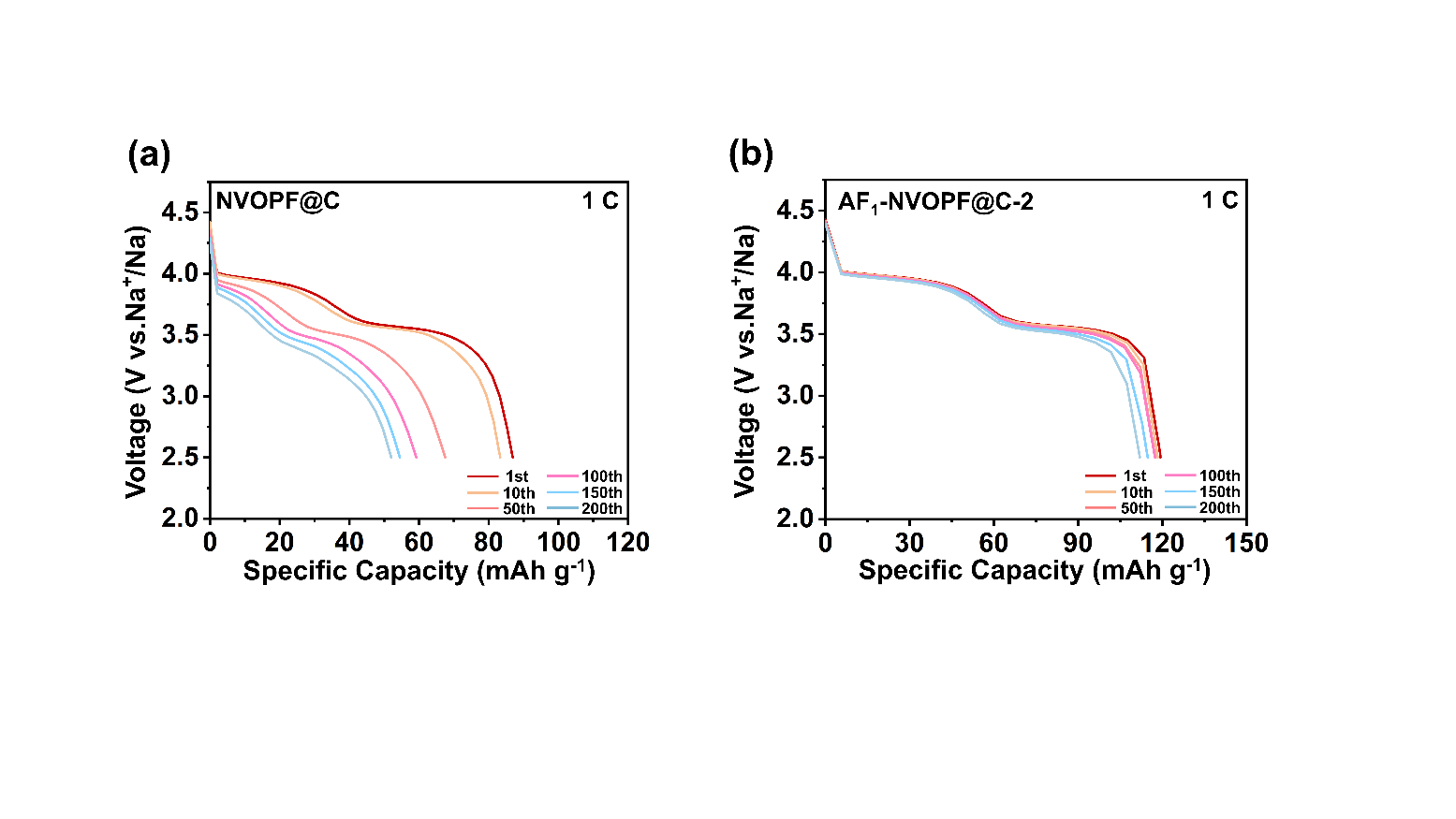


**Figure S11.** Discharge curves at different cycles of a) NVOPF@C and b) AF_1_-NVOPF@C-2.


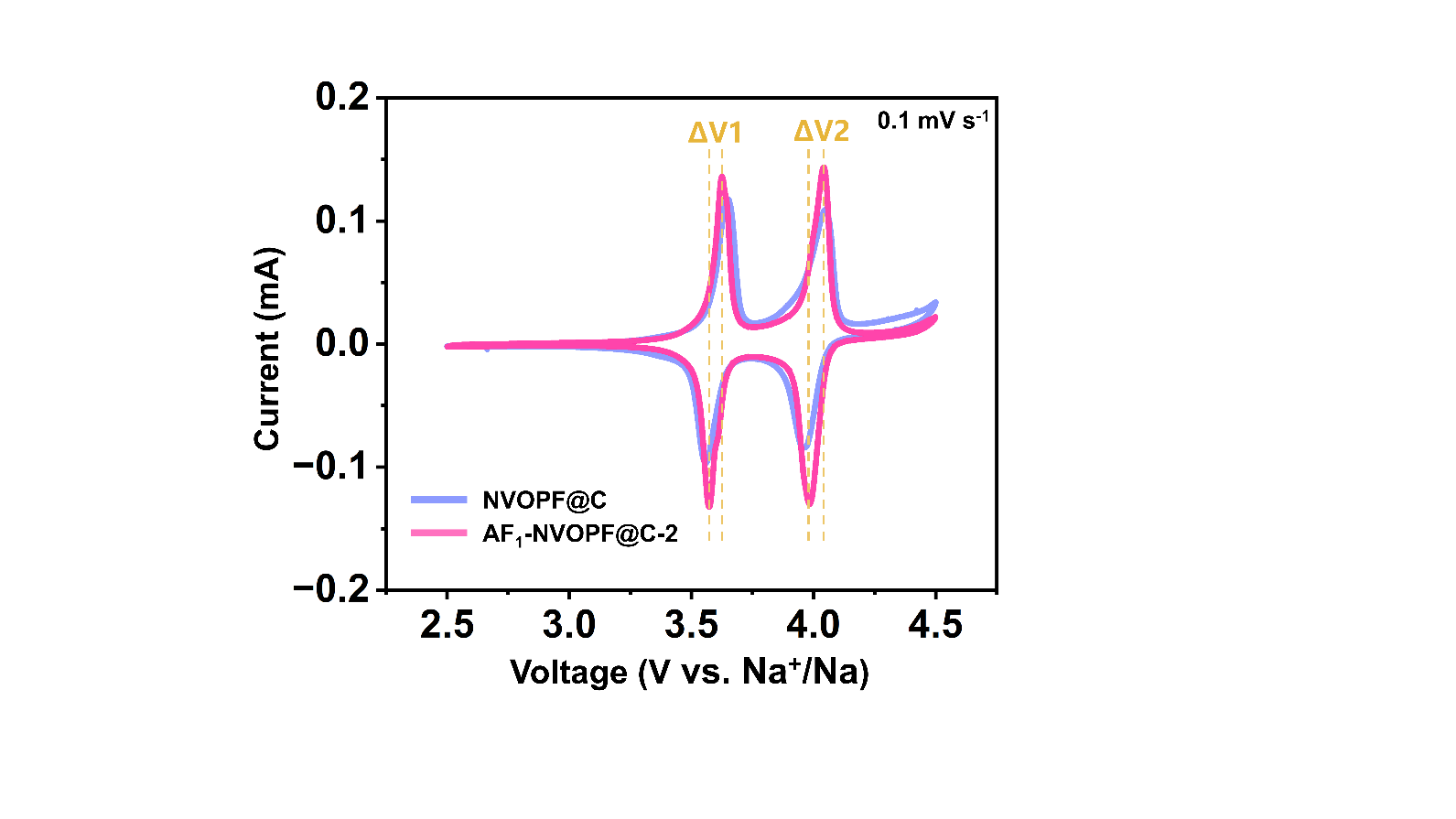


**Figure S12.** CV curves of the first cycle at 0.1 mV s^−1^ for NVOPF@C and AF_1_-NVOPF@C-2.

**
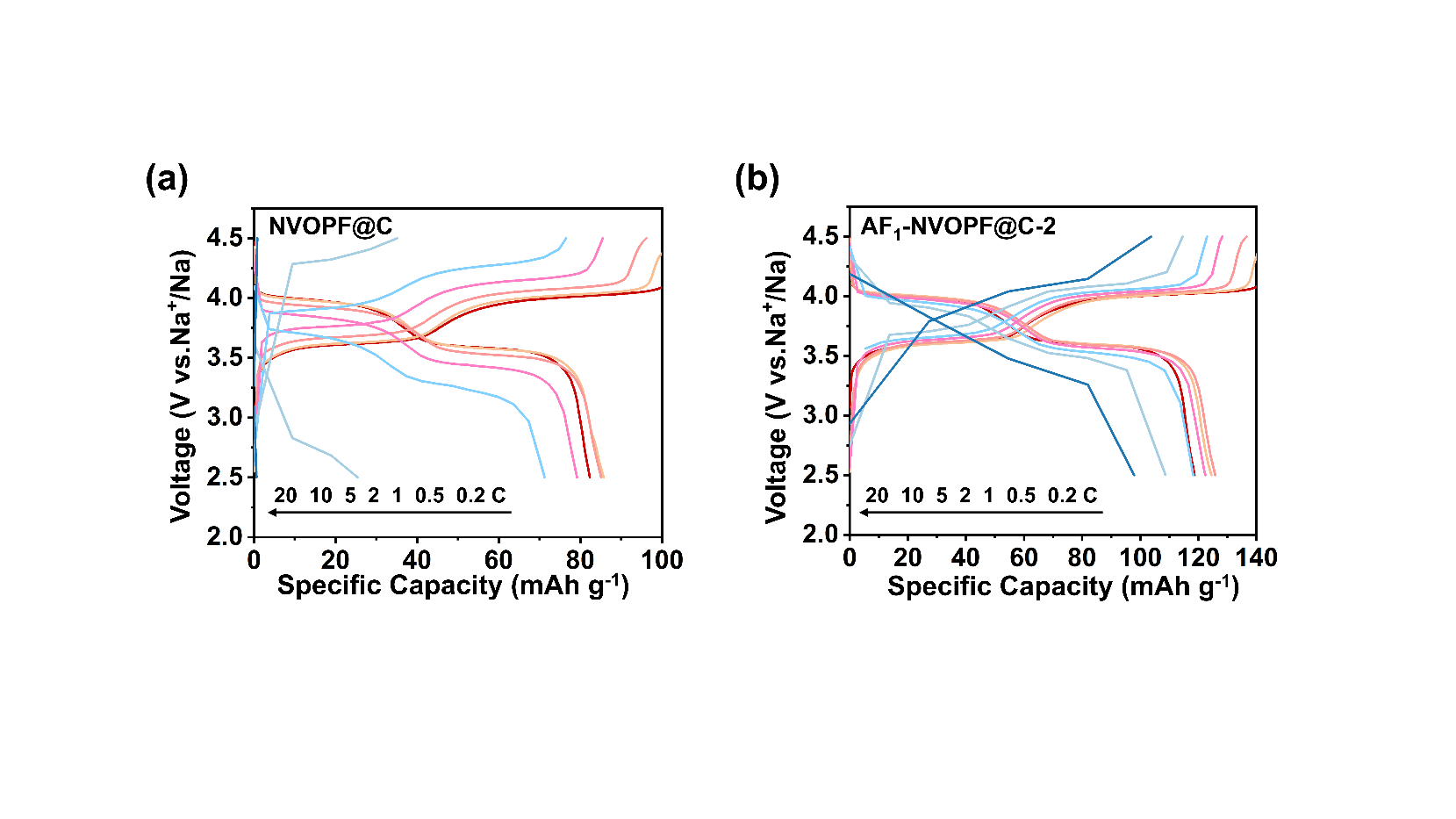
**

**Figure S13.** The GCD profiles at various current densities of a) NVOPF@C and b) AF_1_-NVOPF@C-2.


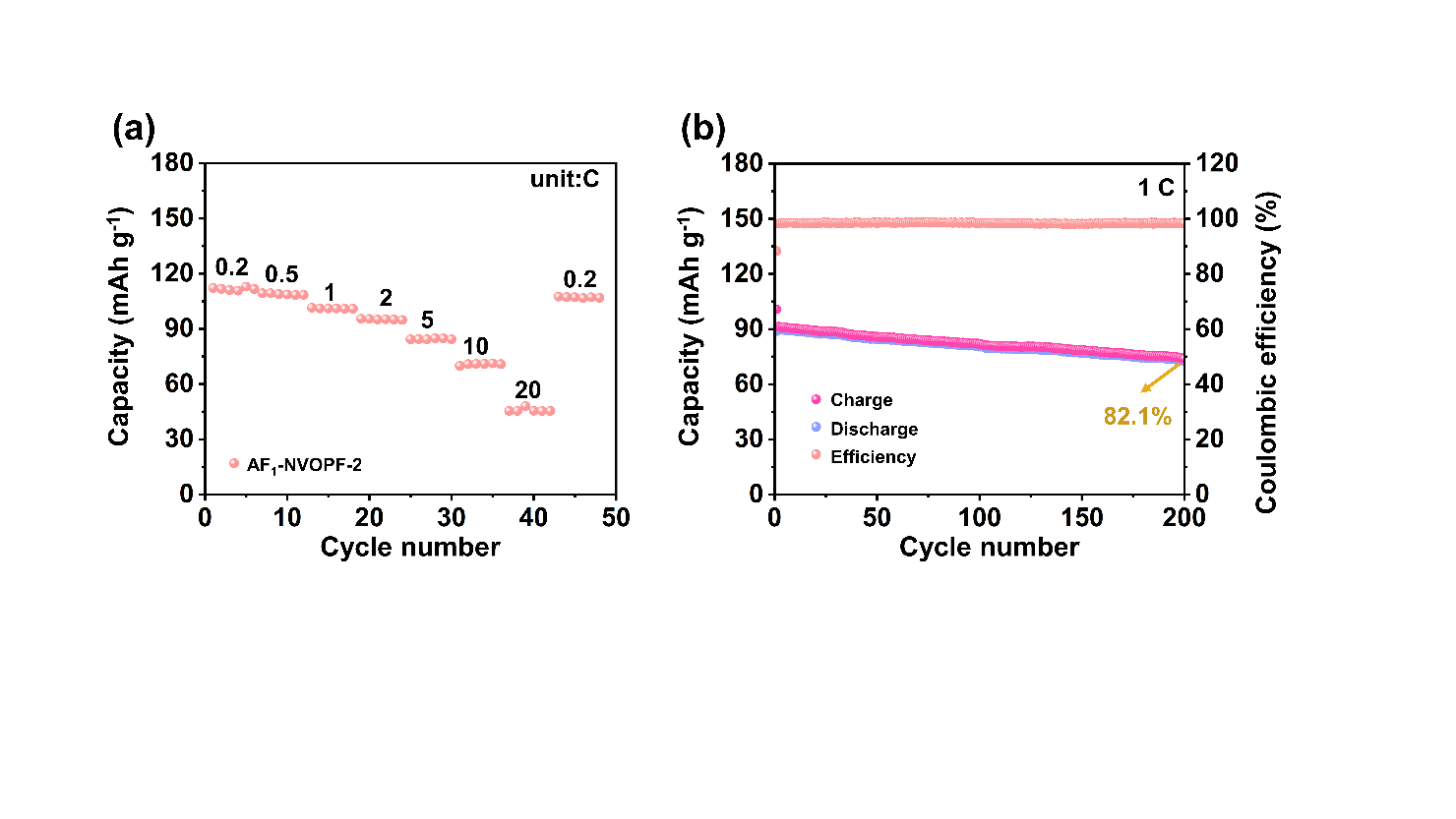


**Figure S14**. a) Rate capability from 0.2 C to 20 C and b) cycling stability at 1 C of AF_1_-NVOPF-2.


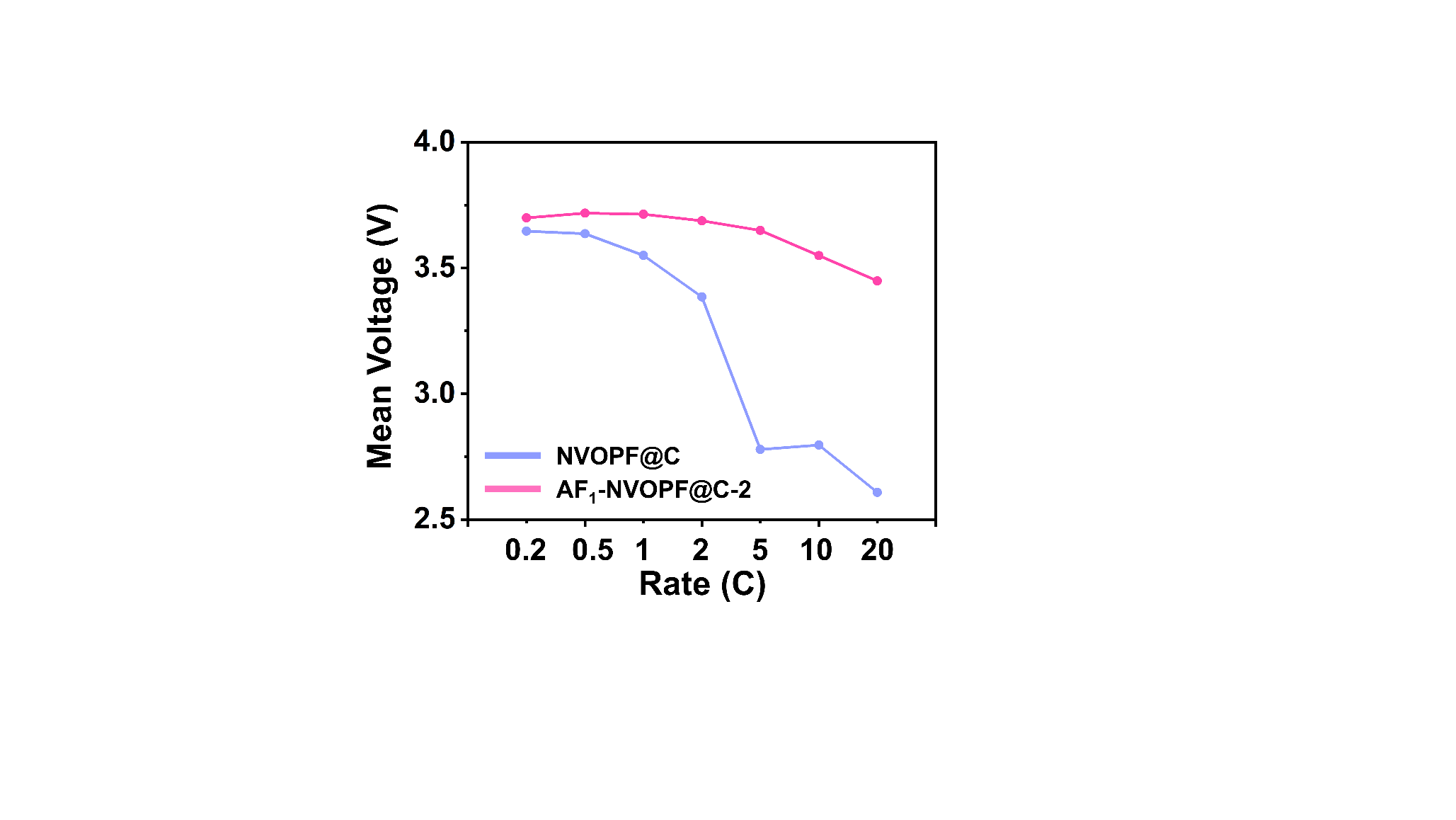


**Figure S15.** Mean voltage of NVOPF@C and AF_1_-NVOPF@C-2 at various current densities.

**
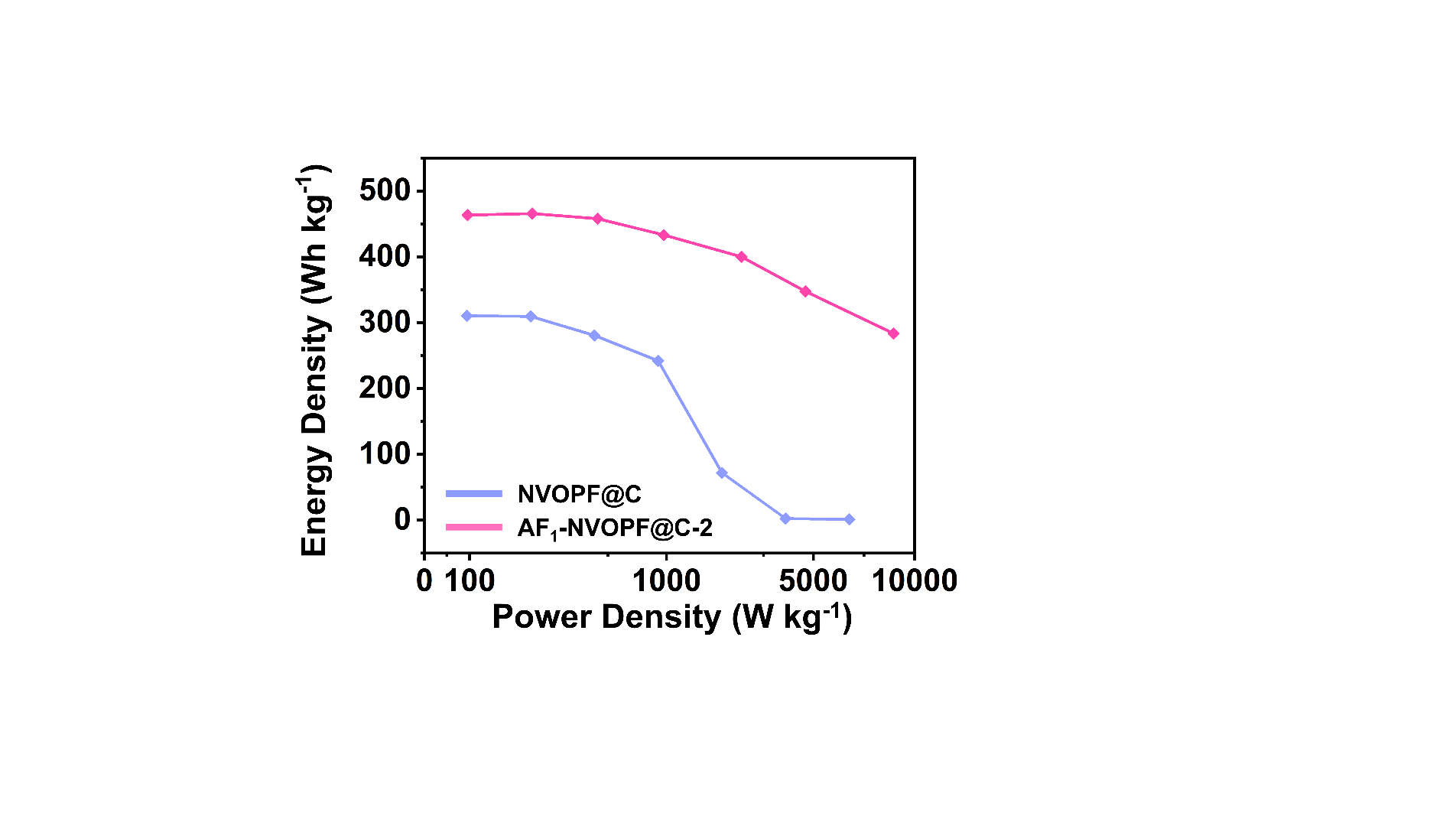
**

**Figure S16.** Power and energy densities of NVPOF@C and AF_1_-NVOPF@C-2.

**
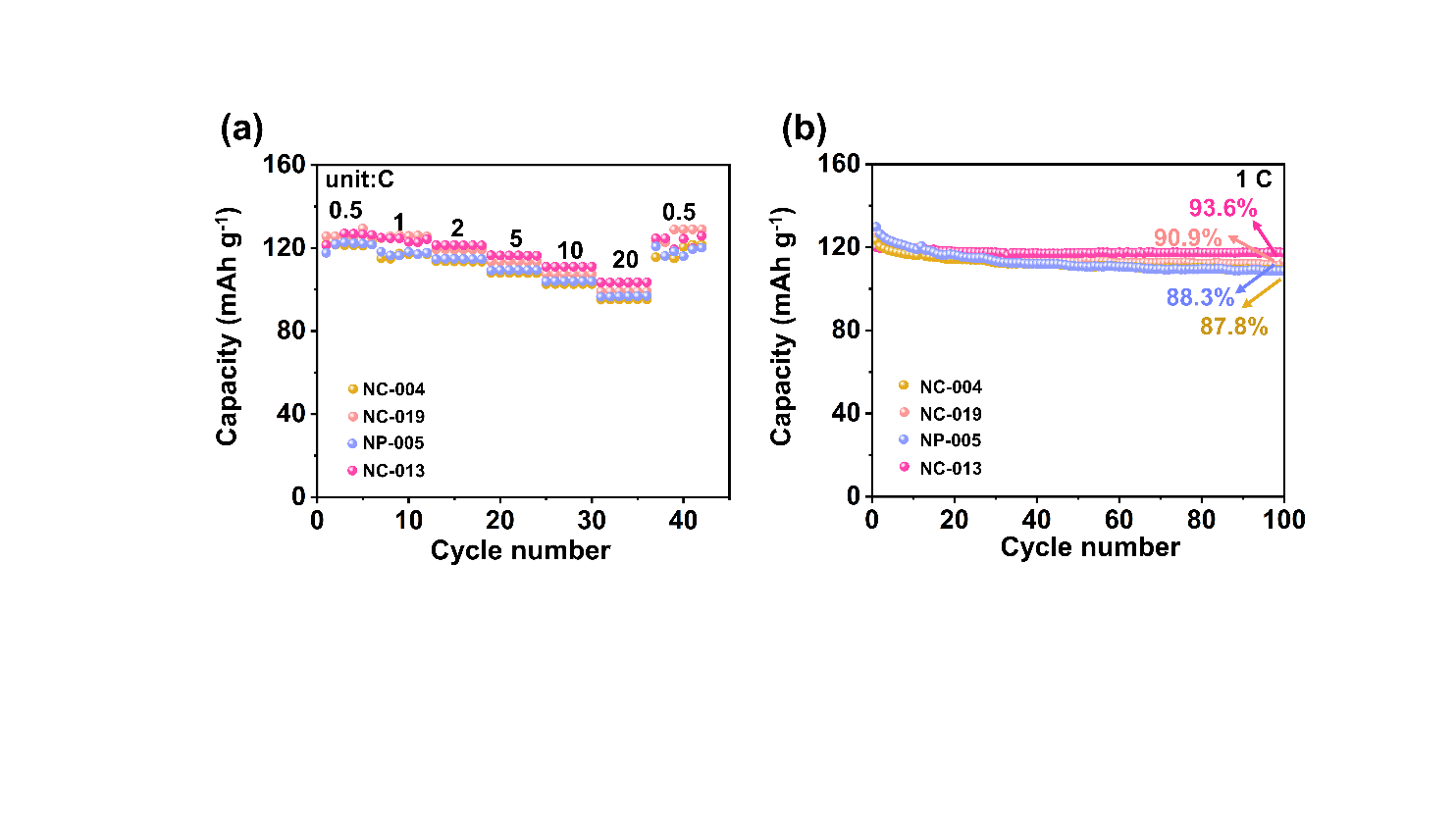
**

**Figure S17.** a) Rate capability from 0.5 C to 20 C and b) cycling stability at 1 C of AF_1_-NVOPF@-2 in different electrolyte systems (NC-004 (1M NaClO_4_ in EC: PC=1:1 Vol% with 5% FEC), NC-019 (1M NaClO_4_ in EC: DMC=1:1 Vol% with 5% FEC), NP-005 (1M NaPF_6_ in DIGLYME) and NC-013 (1M NaClO_4_ in PC with 5% FEC).


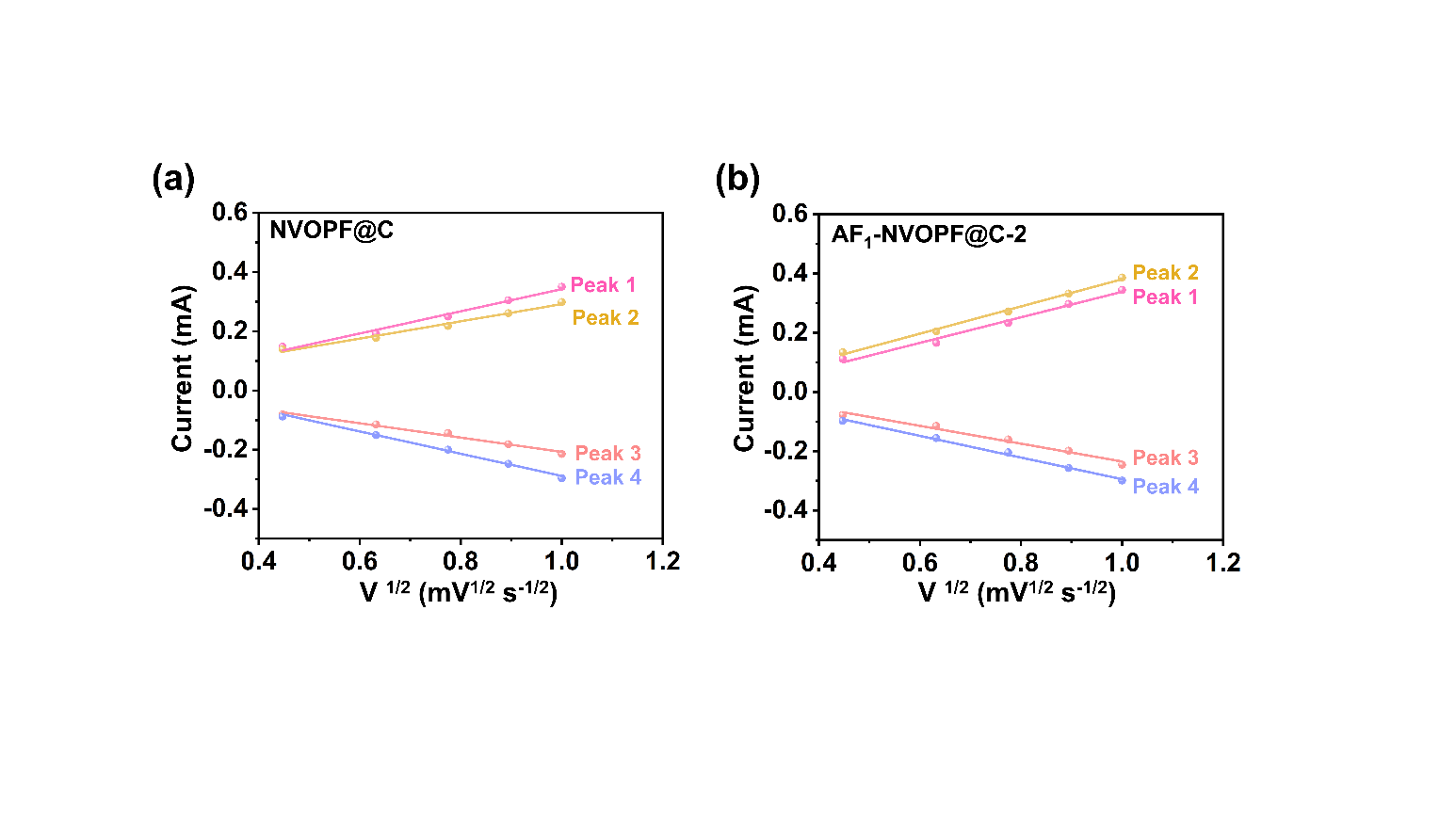


**Figure S18.** Fitting curve of the square root of peak current (*i*_p_) and sweep rate (*v*^1/2^) in a) NVOPF@C and b) AF_1_-NVOPF@C-2.

**Calculation process for the *D*_Na_ values through CV tests:**

The above peaks show the linear relationship between the peak current (*i*_p_) and the square root of the scan rate (*v*^1/2^), which implies that the electrochemical process of NVOPF-based cathodes is diffusion dominant as the intercalated compound. Then, the corresponding diffusion coefficients of Na^+^ (*D*_CV_) of the prepared NVOPF@C and AF_1_-NVOPF@C-2 cathodes can be calculated by the Randles-Sevcik **equation S1**：

$i_{p}=\left( 2.69\times{10}^{5} \right)n^{3/2}AC_{o}D^{1/2}v^{1/2}$ **(S1)**

where n represents the number of electrons transferred in the redox event, and the A indicates the surface area of the NVOPF-based cathode, C_0_ represents the concentration of Na^+^ participates in the electrochemical reaction within the electrolyte, respectively.


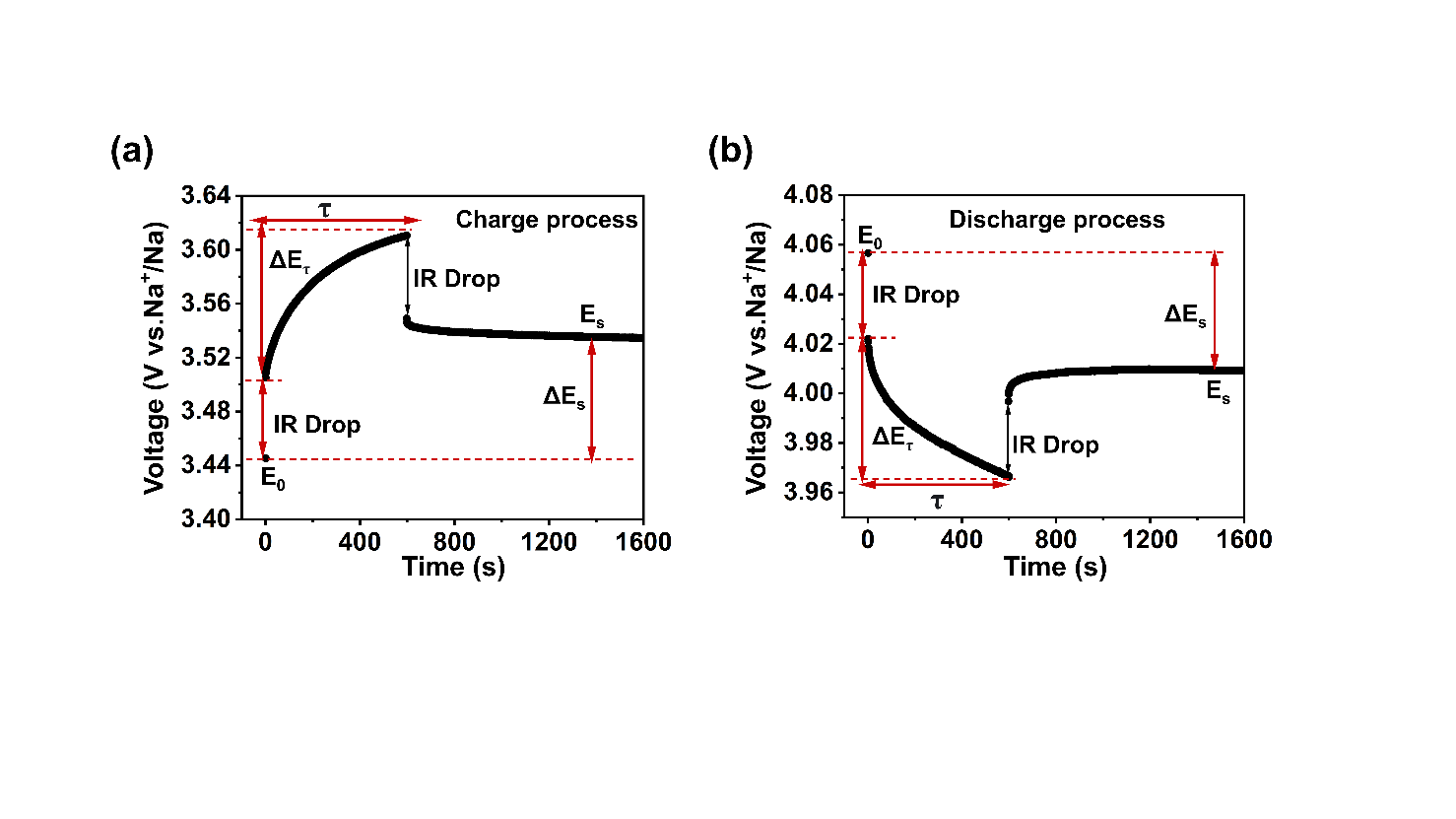


**Figure S19.** a) Schematic diagram of a single GITT titration step during the charging process and b) discharging process of AF_1_-NVOPF@C-2.

**Calculation process for the** ***D*_app, Na_ values through GITT tests:**

The *D*_app, Na_ of as-prepared cathodes are calculated from the GITT potential profiles using Fick’s second law with the following **equation S2**:

$D_{{Na}^{+}}={\frac{4}{\pi\tau}(\frac{m_{B}V_{m}}{M_{B}S})}^{2}{(\frac{{\Delta E}_{s}}{{\Delta E}_{\tau}})}^{2} (\tau\ll\frac{l^{2}}{D_{{Na}^{+}}})$  **(S2)**

where m_B_, M_B_, and V_m_ are the mass, molecular weight, and molar volume of the NVOPF-based materials, respectively; τ is the time for an applied galvanostatic current; S is the active surface of the electrode; l is the average radius of the material particles; and ΔEs and ΔEτ are the quasi-equilibrium potential and the change of cell voltage E during the current pulse, respectively.


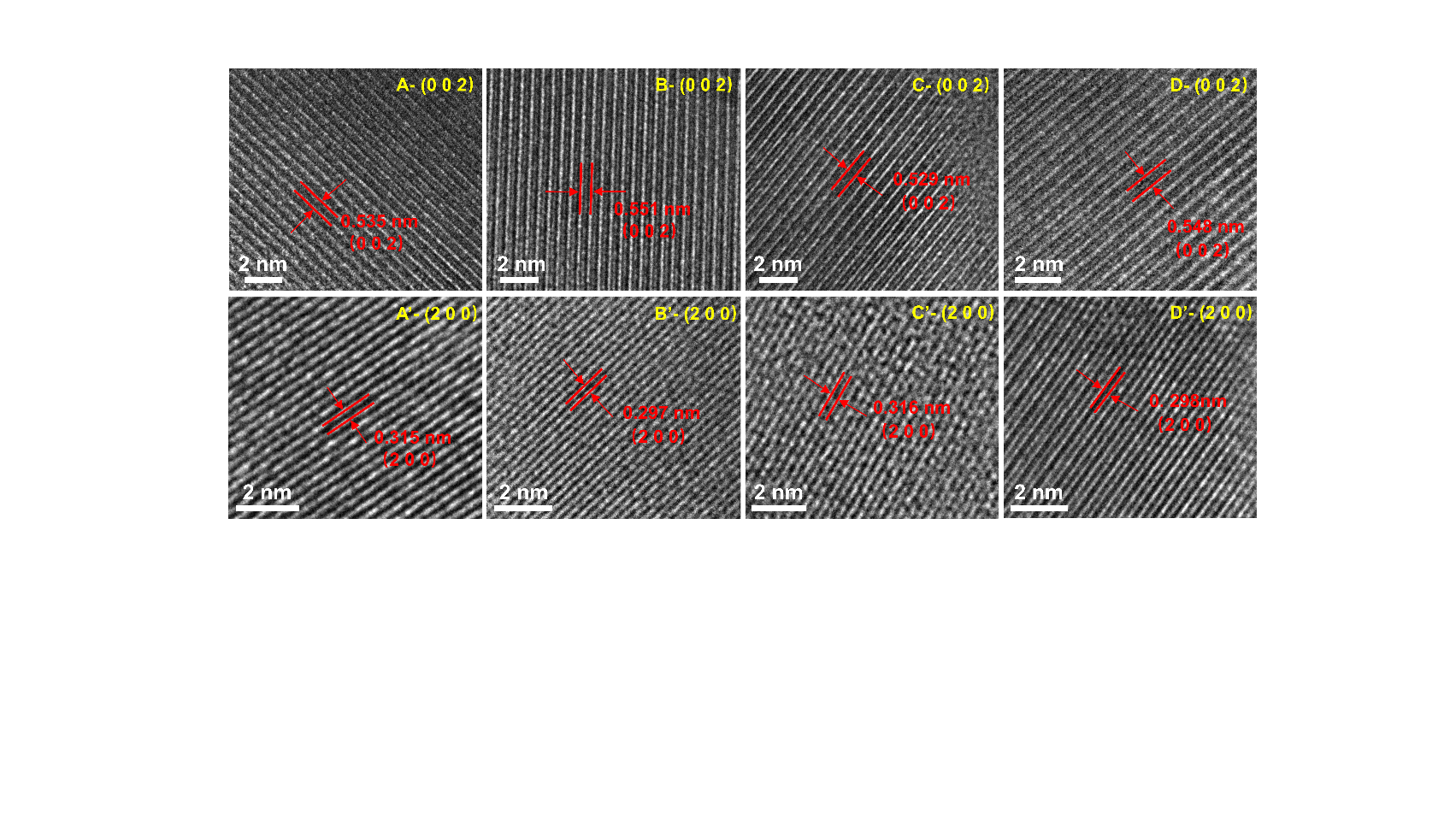


**Figure S20.** Ex-situ HRTEM images of the AF_1_-NVOPF@C-2 at four different states: A is the pristine state with Na_3_V_2_O_2_(PO_4_)_2_F; B is the first fully charged state with NaV_2_O_2_(PO_4_)_2_F; C is the first fully discharged state with Na_3_V_2_O_2_(PO_4_)_2_F; and D is the second fully charged state with NaV_2_O_2_(PO_4_)_2_F.


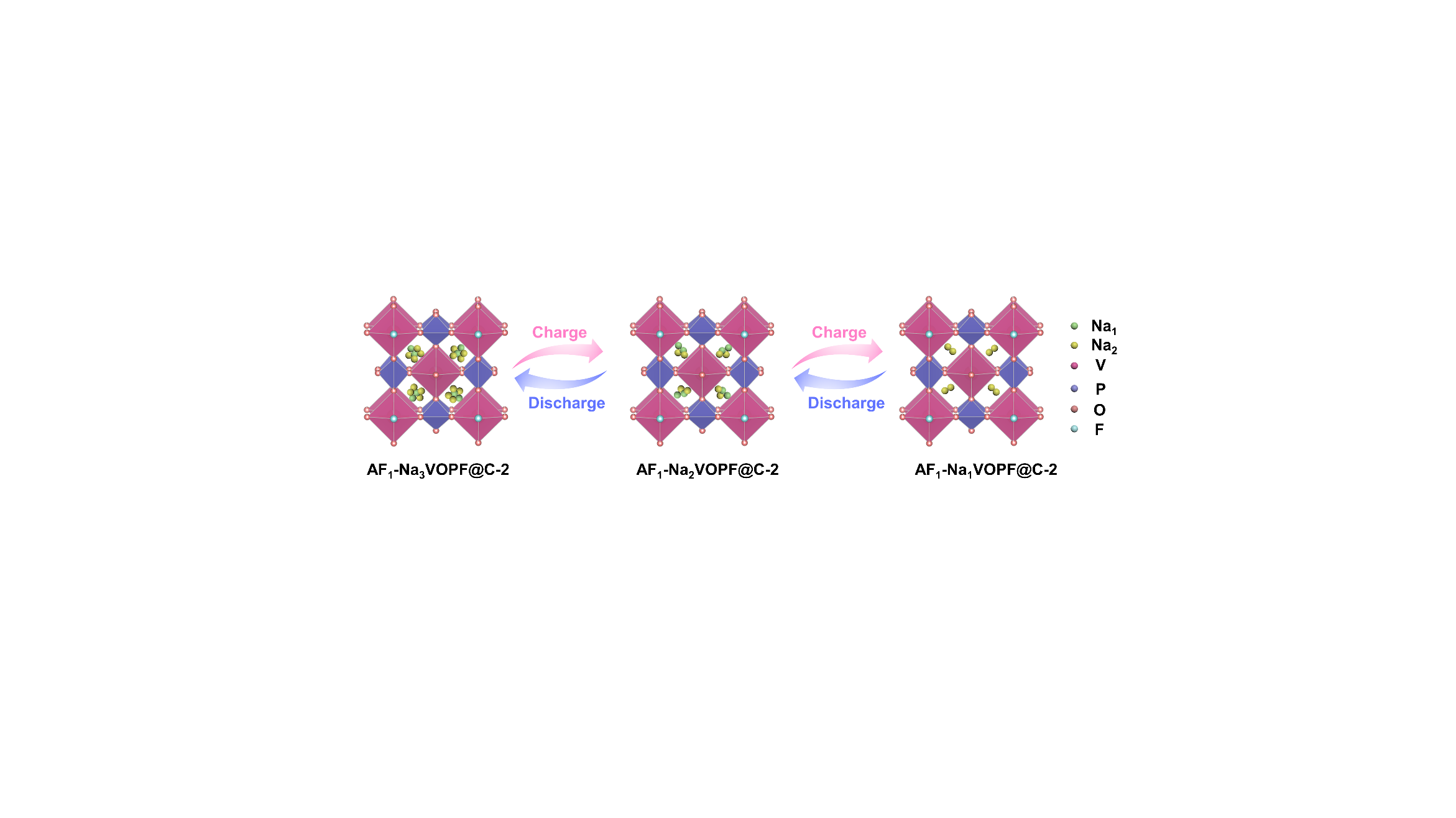


**Figure S21.** Schematic evolution of sodium ion insertion and extraction microstructure.


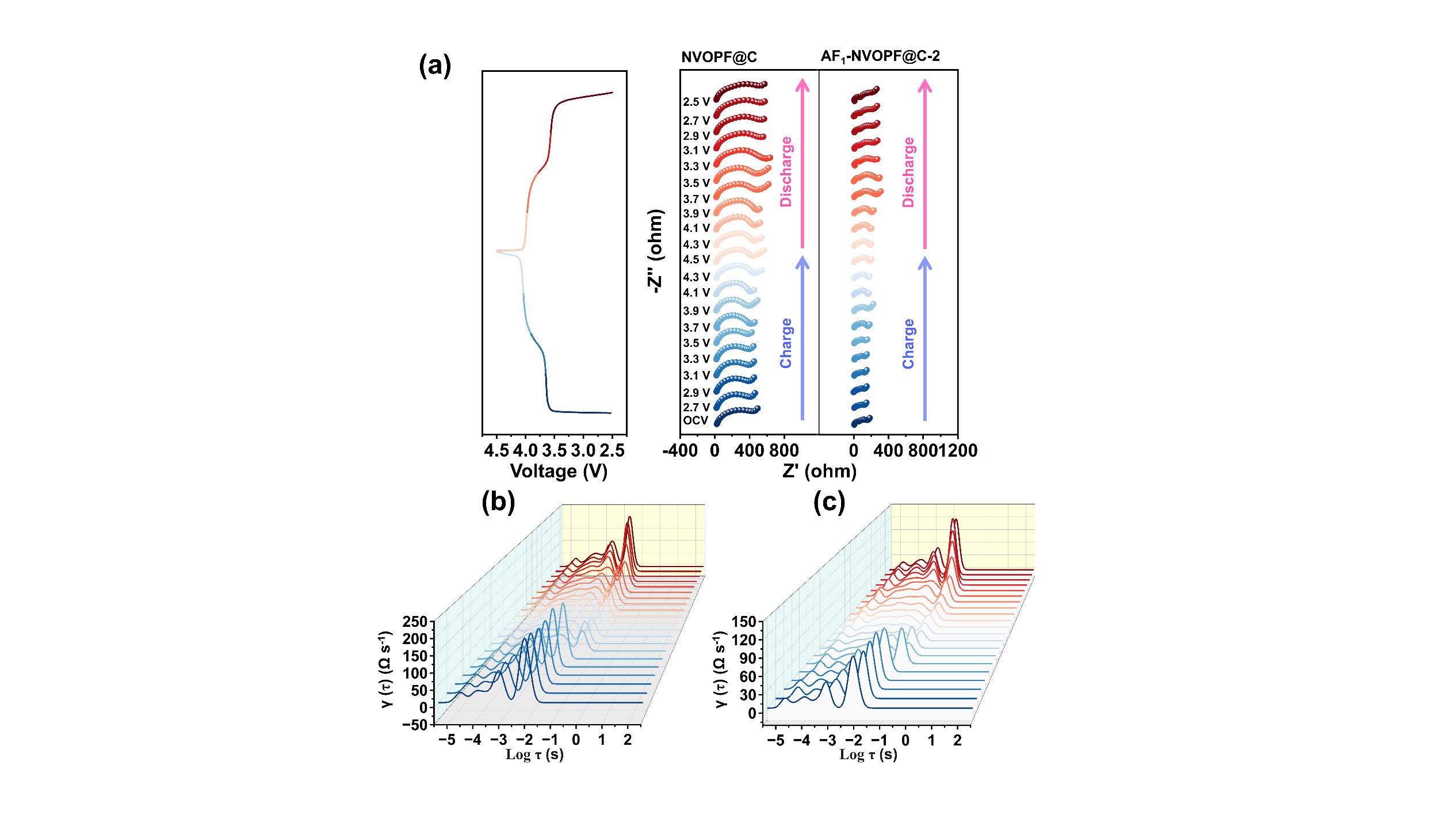


**Figure S22.** a) In-situ EIS spectrums from 2.5 V to 4.5 V and b, c) corresponding DRT analyses curves of NVOPF@C and AF_1_-NVOPF@C-2 at 55 °C.


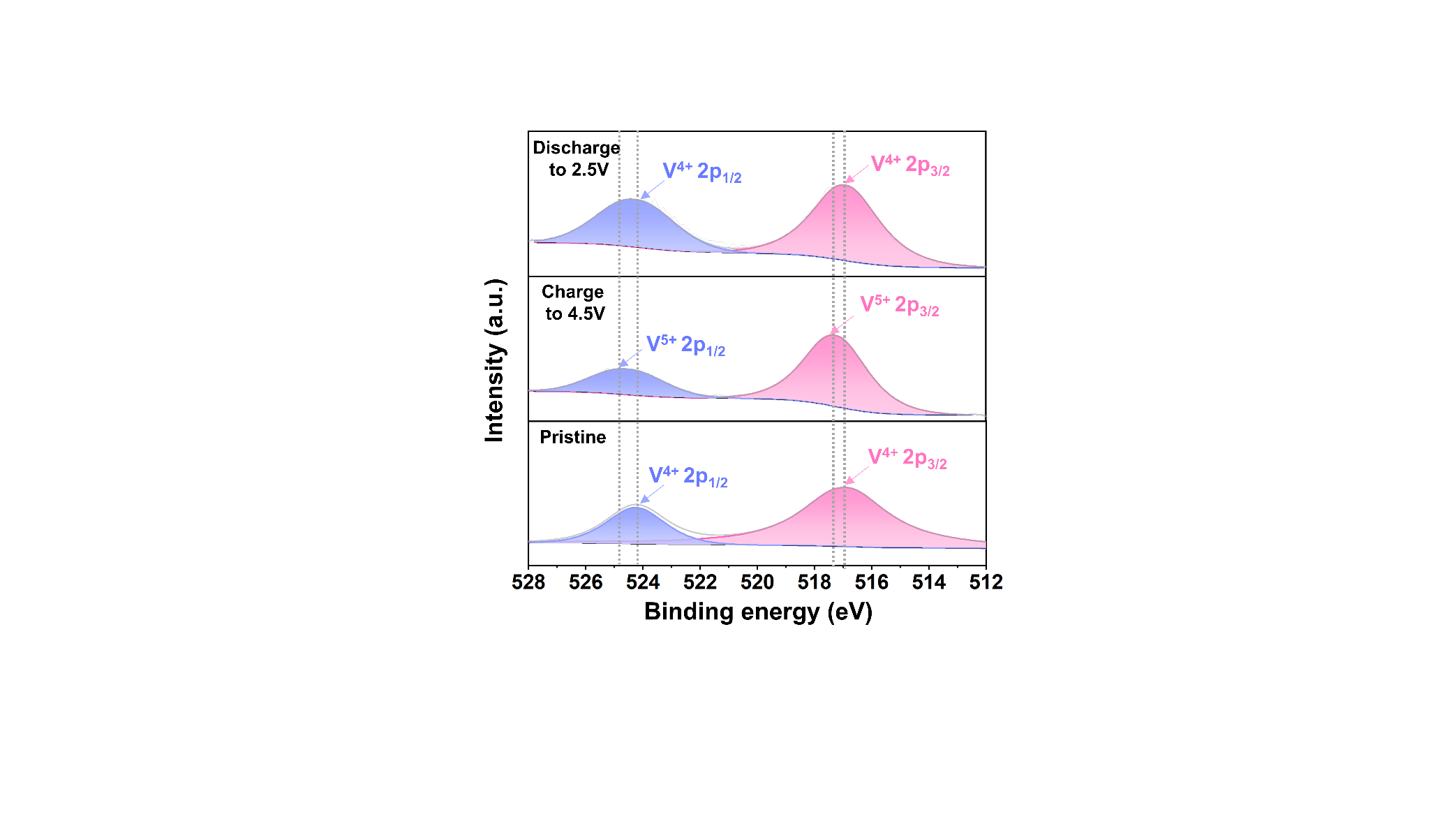


**Figure S23.** High-resolution V 2p spectra of AF_1_-NVOPF@C-2 at the pristine, charge to 4.5 V and discharge to 2.5 V states.


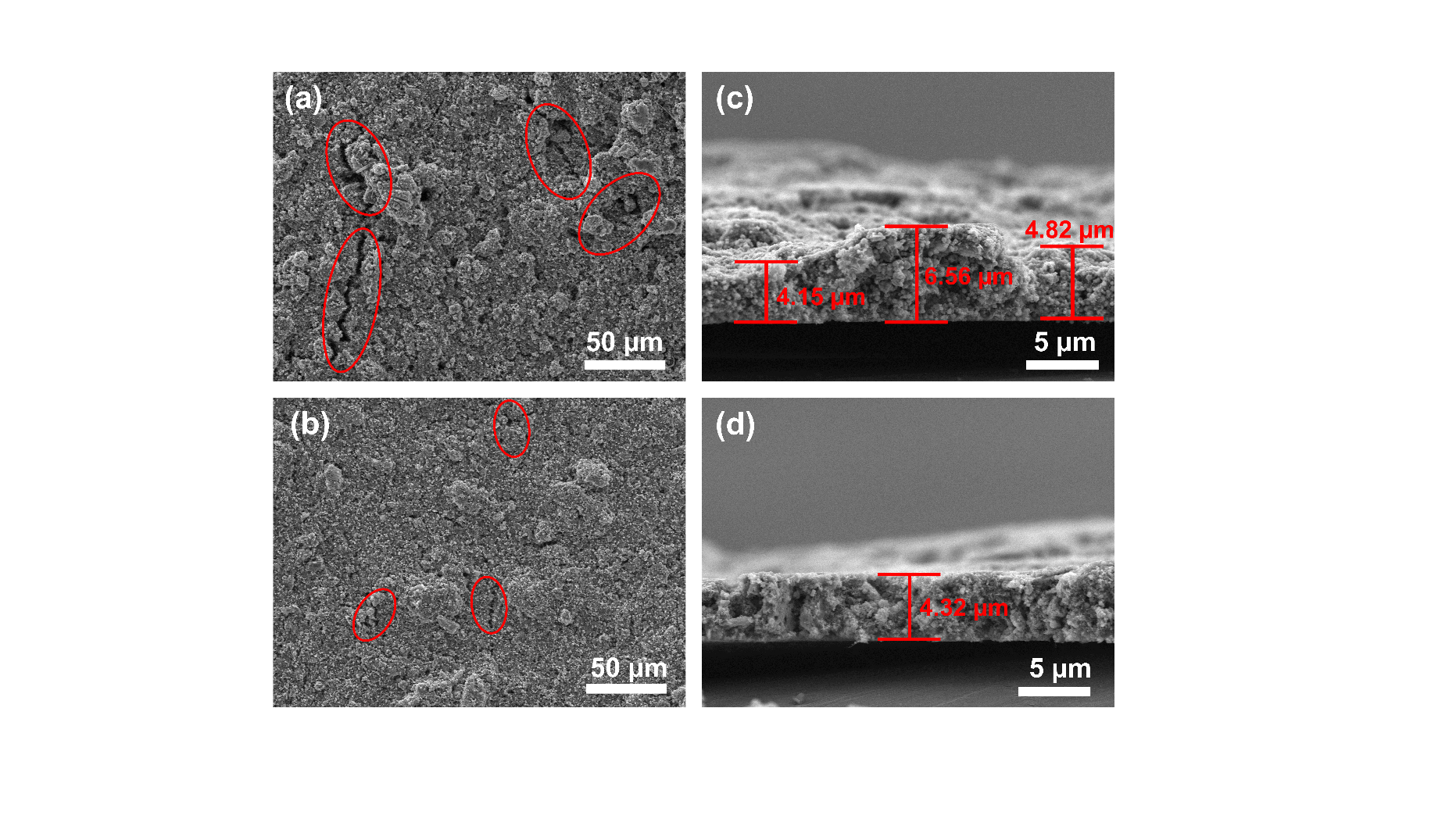


**Figure S24.** SEM images of the surface of the pole piece and the cross sections of the pole pieces of a, c) NVOPF@C and b, d) AF_1_-NVOPF@C-2 after 1000 cycles at 55 °C with a current density of 10 C.


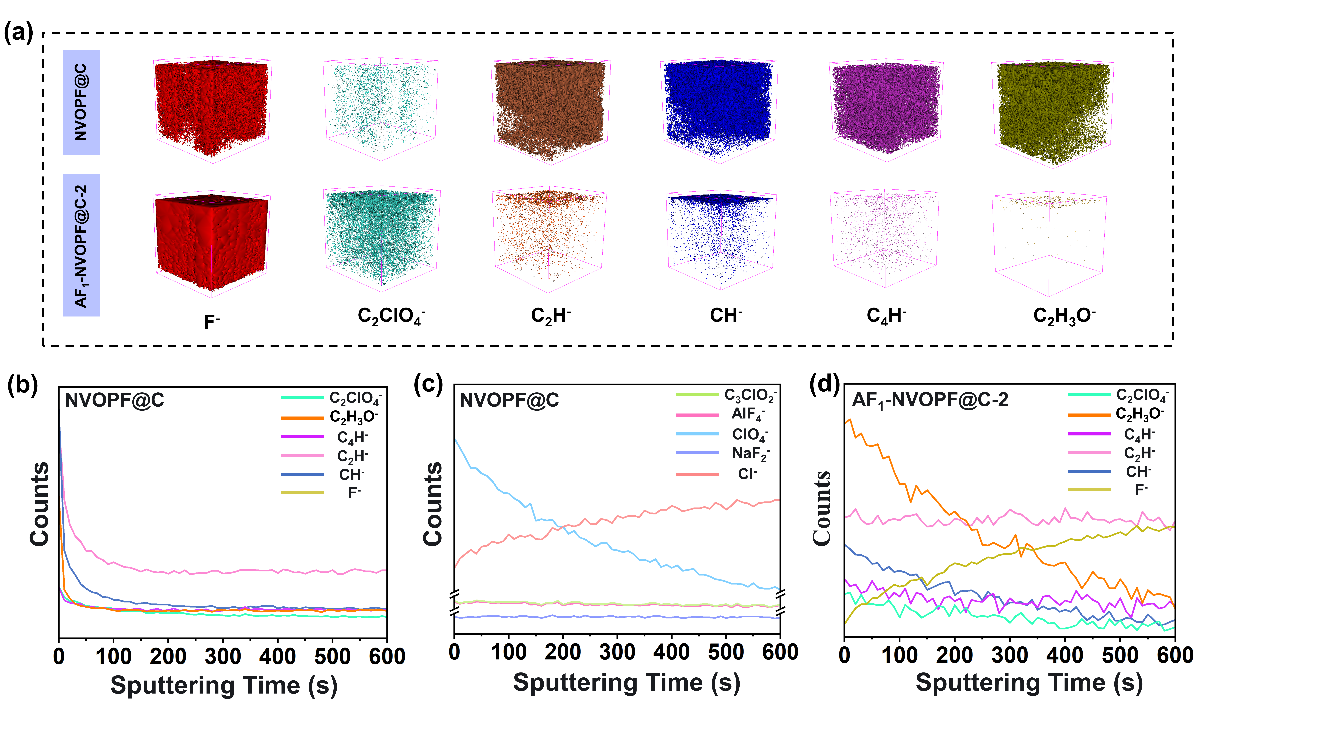


**Figure S25.** Interfacial chemistry of NVOPF@C and AF_1_-NVOPF@C-2. a) TOF-SIMS 3D-mapping images of several representative secondary ion fragments obtained from the cathodes after cycling; b, c and d) depth profiles of several representative secondary ion fragments obtained from the cathodes based on corresponding TOF-SIMS results.


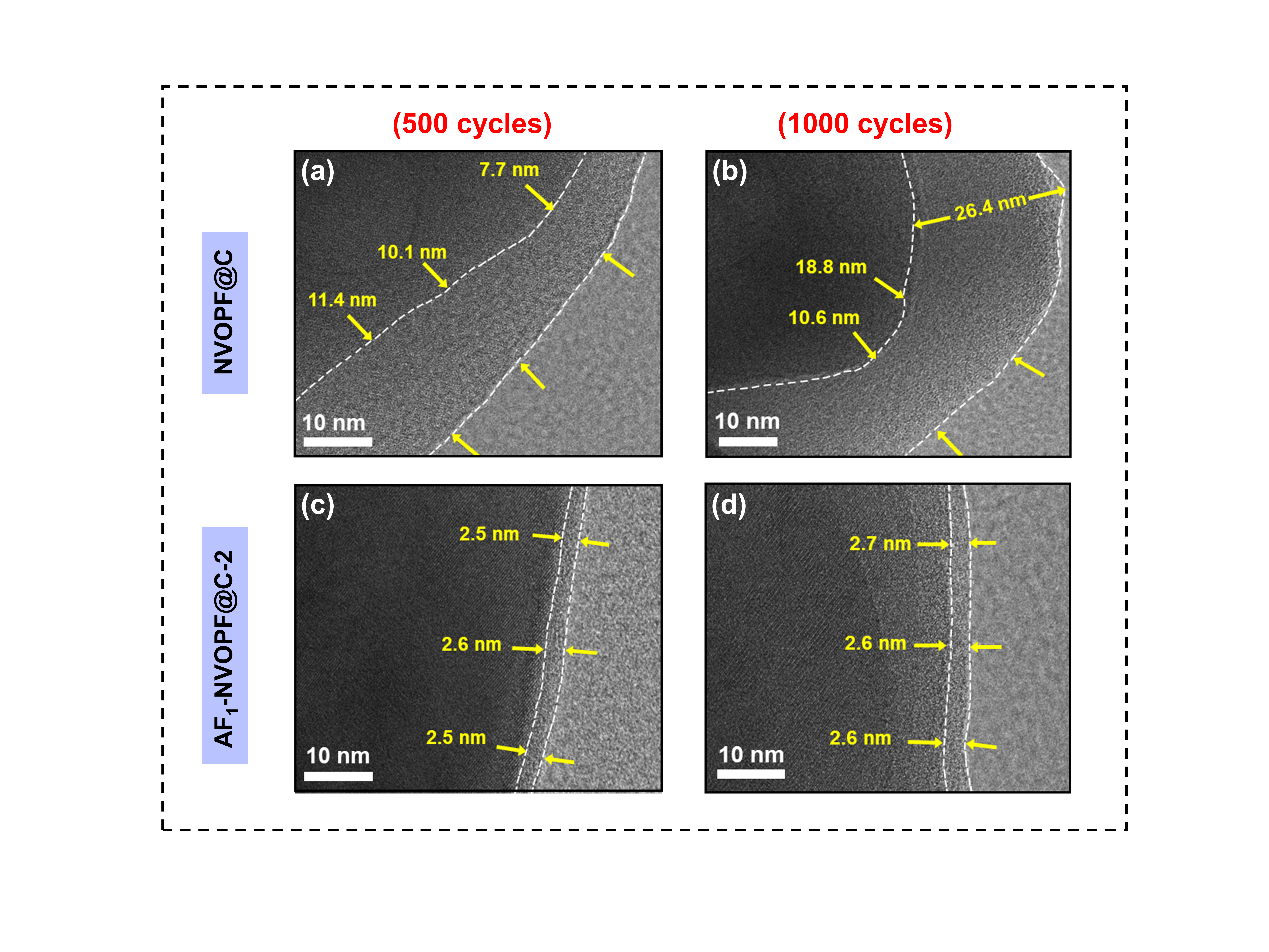


**Figure S26.** HRTEM images of CEI layer at 55 °C for a, b) NVOPF@C and c, d) AF_1_-NVOPF@C-2 after 500 and 1000 cycles.

**
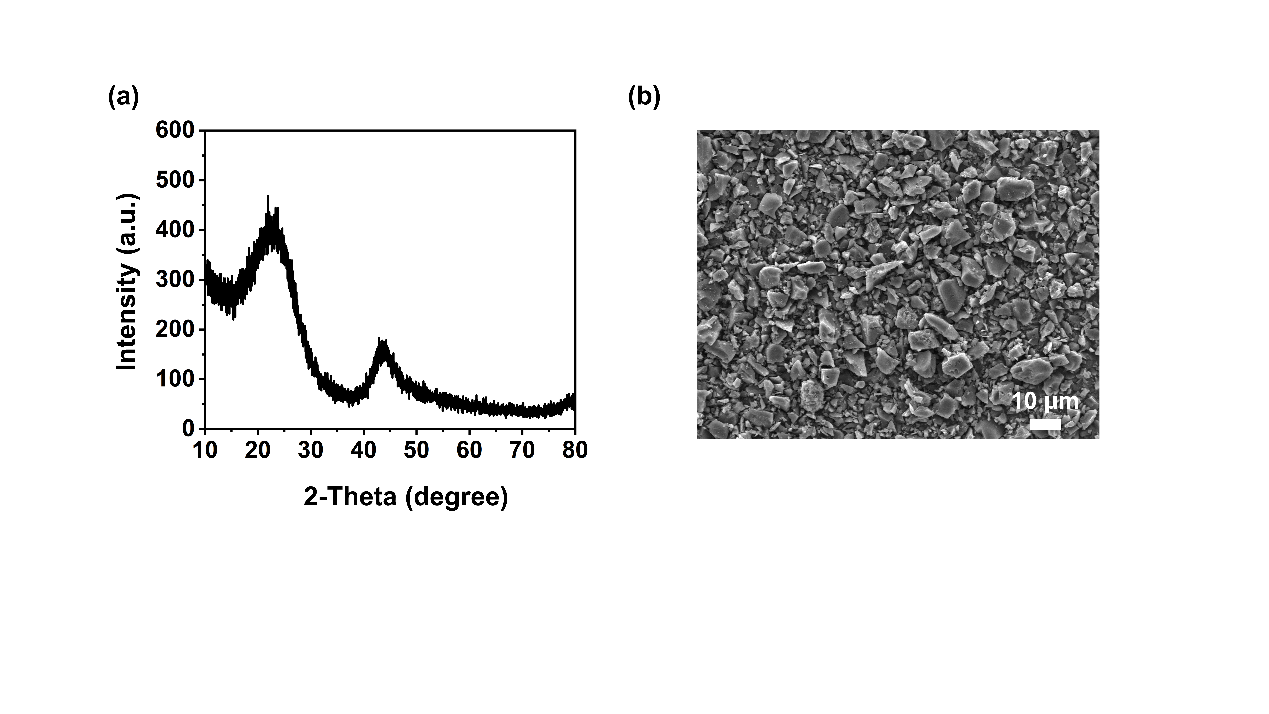
**

**Figure S27.** a) XRD pattern and b) SEM image of the HC anode material.


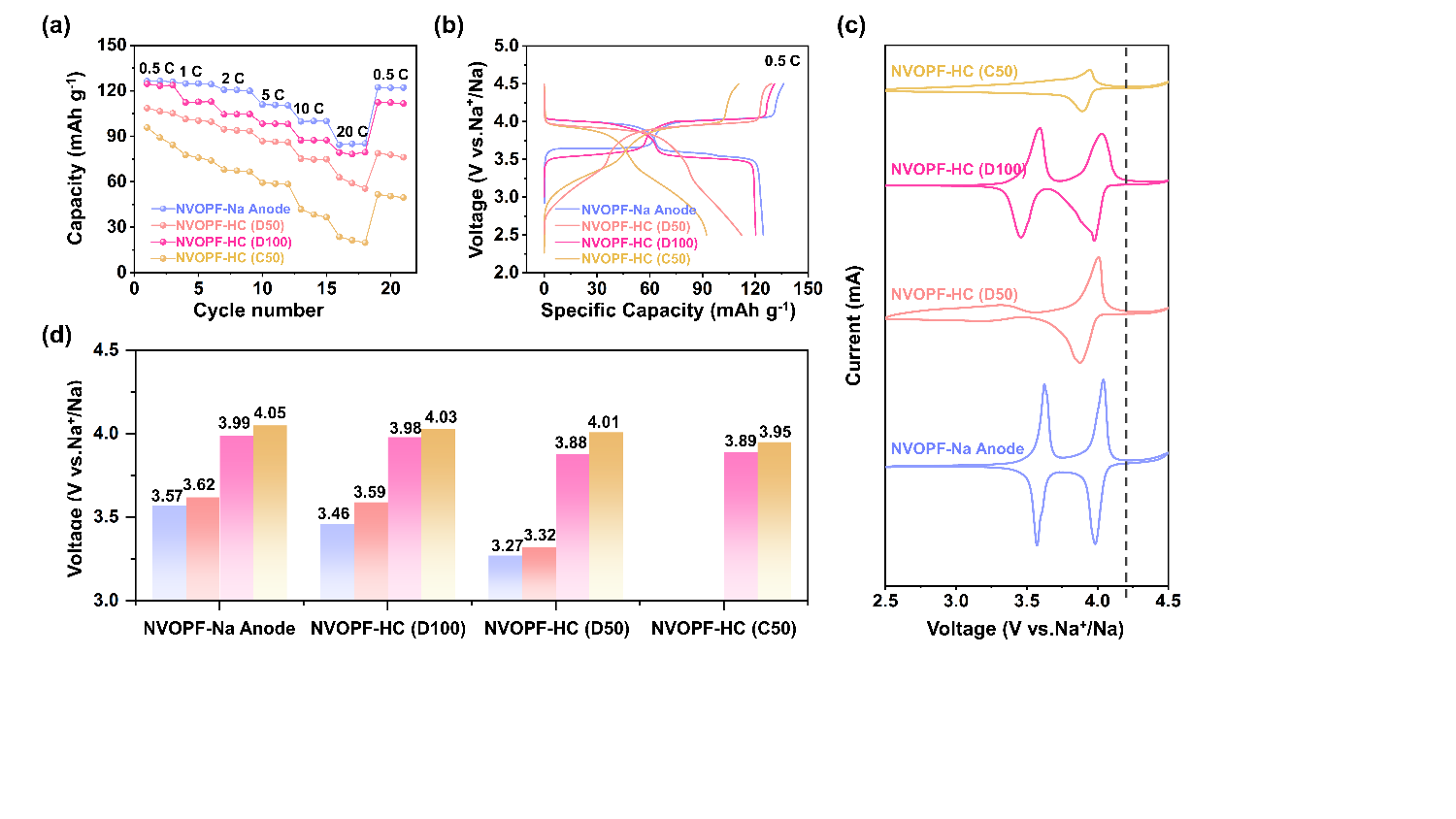


**Figure S28**. Electrochemical performance of full cells with various pretreated HC samples as anodes together with the AF_1_-NVOPF@C-2 cathode. a) Rate capability at 0.5 to 20 C. b) The GCD profiles at 0.5 C. c) CV curves at 0.1 mV s^-1^. d) Statistics of oxidation/reduction peak potentials.

HC was pretreated in half cells via galvanostatic charge/discharge, and discharged to 50% (D50), 100% (D100) of its total capacity, as well as discharged and then re-charged back to 50% (C50) of its total capacity. Subsequently, Na-ion full cells were assembled using the variously pretreated HC samples as anodes together with the AF_1_-NVOPF@C-2 cathode. As shown in Figure S28a, the full cell employing AF_1_-NVOPF@C-2 as the cathode and HC (D100) as the anode delivered the best rate performance. Moreover, its charge/discharge profiles at 0.5 C (Figure S28b) and CV curves (Figure S28c), and redox peaks (Figure S28d) were closest to those of the AF_1_-NVOPF@C-2//Na half-cells. The superior electrochemical performance of the AF_1_-NVOPF@C-2//HC (D100) full cell can be attributed to the fact that HC (D100) provides sacrificial Na^+^ for SEI formation and compensates for irreversible capacity loss caused by Na^+^ trapping in hard carbon. Therefore, pre-sodiated HC (D100) was selected as the anode in full cells to ensure the excellent electrochemical performance.


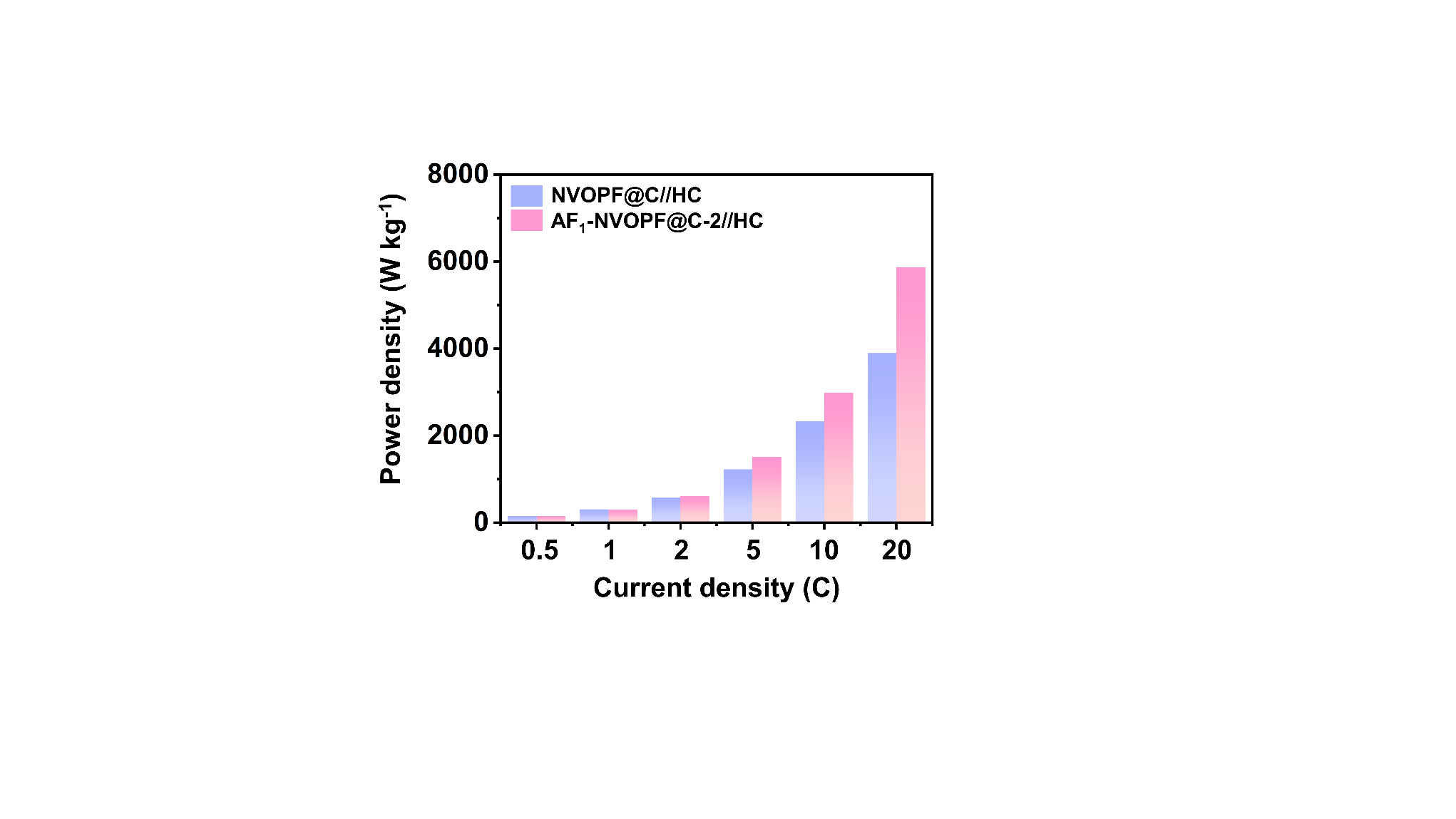


**Figure S29.** Power densities at different current densities of AF_1_-NVOPF@C-2//HC and NVOPF@C//HC.

**Table S1.** The Al content for pH=7 material with 1% AlF_3_ coating amount measured by ICP-OES.

| Sample | Element | Content(%) |
| --- | --- | --- |
| AF_1_-NVOPF@C-2 | Al | 0.7% |

**Table S2.** Voltage difference of the first cycle in CV curves at a scan rate of 0.1 mV s^−1^ at 55°C.

| Cathode | Voltage difference (mV) | |
| --- | --- | --- |
| AF_1_-NVOPF@C-2 | △V_1_=50 | △V_2_=61 |
| NVOPF@C | △V_3_=96 | △V_4_=89 |

**Table S3.** A comparison of the high-temperature performance of AF_1_-NVOPF@C-2 with other reported cathodes for SIBs.

| Cathodes | C-rate capability | Capacity retention | Temperature | Ref. |
| --- | --- | --- | --- | --- |
| **AF_1_-NVOPF@C-2** | **128.3 mAh g**^−1^**@ 0.2 C**  **84.4 mAh g**^−1^**@ 20 C** | **10 C, 1000 cycles (84.9%)** | **55 ℃** | **This work** |
| NVTC | 108 mAh g^−1^@ 1 C  94.3 mAh g^−1^@ 20 C | 20 C, 910 cycles (80%) | 50 ℃ | [2] |
| NVOPF | 118 mAh g^−1^@ 0.2 C  80 mAh g^−1^@ 30 C | 0.2 C, 200 cycles  (81.72%) | 55 ℃ | [3] |
| NVPF | 118.2 mAh g^−1^@ 0.2 C  53.85 mAh g^−1^@ 20 C | 2 C, 100 cycles  (33.8%) | 60 ℃ | [4] |
| NVPF@C@CMC | 122.8 mAh g^−1^@ 0.2 C  55.8 mAh g^−1^@ 20 C | 5 C, 300 cycles  (73.9%) | 45 ℃ | [5] |
| NFVPF | 97 mAh g^−1^@ 0.5 C  60 mAh g^−1^@ 20 C | 10 C, 150 cycles  (87.6%) | 50 ℃ | [6] |

**References:**

1. H. Zhou, Z. T. Cao, Y. F. Zhou, J. X. Li, Z. H. Ling, G. Z. Fang, S. Q. Liang, X. X. Cao, Unlocking rapid and robust sodium storage of fluorophosphate cathode via multivalent anion substitution. *Nano Energy* **2023**, *114*, 108604.
2. X. Y. Wang, Z. X. Sun, W. Lv, Z. H. Zhan, M. Huang, Q. Wang, F. Zhang, H. Wang, X. J. Liu, Advanced Multifunctional Sodium-Ion Battery with High Current Conversion, Long Cycle Life, and All-Climate Temperature Range by Dual-Multivalent Cation Doping Strategy. *Adv. Energy Mater.* **2025**, *15*, 2500471.
3. X. X. Zhao, Z. Y. Gu, W. H. Li, X. Yang, J. Z. Guo, X. L. Wu, Temperature-Dependent Electrochemical Properties and Electrode Kinetics of Na_3_V_2_(PO_4_)_2_O_2_F Cathode for Sodium-Ion Batteries with High Energy Density. *Chem. Eur. J.* **2020**, *26*, 7823.
4. J. H. wang, K. Matsumoto, R. Hagiwara, Electrolytes toward High-Voltage Na_3_V_2_(PO_4_)_2_F_3_ Positive Electrode Durable against Temperature Variation. *Adv. Energy Mater.* **2020**, *10*, 2001880.
5. K. Liang, H. S. Zhao, J. B. Li, X. B. Huang, Y. R. Ren, High-performance Na_3_V_2_(PO4)_2_F_3_ cathode obtained by a three-in-one strategy for self-sodium compensation, interface modification, and crosslinked carbon coatings. *Appl. Surf. Sci.* **2023**, *615*, 156412.
6. J. M. Dong, J. C. Xiao, Y. F. Yu, J. R. Wang, F. Chen, S. Wang, L. M. Zhang, N. Q. Ren, B. C. Pan, C. H. Chen, Electronic structure regulation of Na_2_FePO_4_F cathode toward superior high-rate and high-temperature sodium-ion batteries. *Energy Storage Mater.* **2022***,* *45*, 851.
